# Targeted thrombolysis by magnetoacoustic particles in photothrombotic stroke model

**DOI:** 10.1186/s40824-022-00298-y

**Published:** 2022-10-22

**Authors:** Wonseok Choi, Hyeyoun Cho, Gahee Kim, Inchan Youn, Jaehong Key, Sungmin Han

**Affiliations:** 1 Biomedical Research Division, Korea Institute of Science and Technology, Seoul, Republic of Korea; 2 Department of Biomedical Engineering, Yonsei University, Wonju, Republic of Korea; 3 Divison of Bio-Medical Science & Technology, Korea Institute of Science and Technology School, Seoul, Republic of Korea; 4 KHU-KIST Department of Converging Science and Technology, Kyung Hee University, Seoul, Republic of Korea

**Keywords:** Thrombolysis, Ischemic Stroke, Photothrombosis, Recombinant Tissue Plasminogen Activator (rtPA), Controlled Release Drug Carriers

## Abstract

**Background:**

Recombinant tissue plasminogen activator (rtPA) has a short half-life, and additional hemorrhagic transformation (HT) can occur when treatment is delayed. Here, we report the design and thrombolytic performance of 3 $\upmu$m discoidal polymeric particles loaded with rtPA and superparamagnetic iron oxide nanoparticles (SPIONs), referred to as rmDPPs, to address the HT issues of rtPA.

**Methods:**

The rmDPPs consisted of a biodegradable polymeric matrix, rtPA, and SPIONs and were synthesized *via* a top-down fabrication.

**Results:**

The rmDPPs could be concentrated at the target site with magnetic attraction, and then the rtPA could be released under acoustic stimulus. Therefore, we named that the particles had magnetoacoustic properties. For the *in vitro* blood clot lysis, the rmDPPs with magnetoacoustic stimuli could not enhance the lytic potential compared to the rmDPPs without stimulation. Furthermore, although the reduction of the infarcts *in vivo* was observed along with the magnetoacoustic stimuli in the rmDPPs, more enhancement was not achieved in comparison with the rtPA. A notable advantage of rmDPPs was shown in delayed administration of rmDPPs at poststroke. The late treatment of rmDPPs with magnetoacoustic stimuli could reduce the infarcts and lead to no additional HT issues, while rtPA alone could not show any favorable prognosis.

**Conclusion:**

The rmDPPs may be advantageous in delayed treatment of thrombotic patients.

**Supplementary Information:**

The online version contains supplementary material available at 10.1186/s40824-022-00298-y.

## Background

Thrombosis is the blockage of a blood vessel by a blood clot. The occlusion of blood vessels supplies abnormal oxygen and nutrients into surrounding occluded tissues, which can induce several diseases, such as ischemic/hemorrhagic stroke, coronary infarction, and pulmonary embolism. Conventional thrombosis therapies mainly consist of mechanical thrombectomy and treatment with intravenous (IV) thrombolytic agents. However, thrombectomy is not only technically limited to specific thrombotic patients but can also cause embolism, vasospasm, and increased traumatic injury in vessels [1–7]. As an alternative to thrombectomy, thrombolytic agents are widely utilized in the clinically field since they can be injected without a particular surgical procedure. However, recombinant tissue plasminogen activator (rtPA), one of the a typical thrombolytic agents, has a short half-life *in vivo* (approximately < 10 min) and, with late treatment, arises hemorrhagic transformation (HT) can still occur [8–11].

Recently, drug carriers have been actively developed to overcome the inherent limitations of rtPA. Improvement of targeting potential in drug carriers through surface modification offers promising lytic potential [12–15]. However, the antibody-augmented approach, for example, still faces the challenges of off-target toxicity, rtPA instability, and low efficacy in targeting performance owing to heterogeneous expression of blood clots [13, 16, 17]. Targeting efficacy can be improved using a physical approach [18–23], such as the use of superparamagnetic iron oxide nanoparticles (SPIONs), which allows for magnetic attraction and is also relatively free from the challenges above. Nonetheless, SPIONs are mainly adopted for hyperthermia applications to maximize the lytic potential [18, 21–23], leading to collateral thermal damage to nontarget biological tissues.

Sonothrombolysis that utilizes ultrasound (US) to mechanically lyse blood clots has been used as an alternative technique for treating thrombotic diseases. US is noninvasive, has high spatial resolution, and can stimulate a deep territory compared to other external stimuli. In particle-mediated sonothrombolysis, the efficacy of sonothrombolysis can be accelerated with microbubbles through a cavitation phenomenon [24–28]. However, the instability of microbubbles and lack of targeting potential [29–31] are still limitations to accurately delivering rtPA to a target site [32, 33]. Recently, polymeric particles have been suggested as alternative carriers for use in sonothrombolysis. Polymer-based drug carriers have high stability *in vivo* compared to lipid-based microbubbles and can be modified to respond to stimuli [34, 35]. The thrombolytic potential of polymeric particles can be achieved by physical destruction through cracks or defects on the surface of the particles, releasing their cargo [36–38].

The safety of rtPA delivery can be improved by tuning the degree of drug exposure allowed by the carriers. Most rtPA-containing carriers have been designed to either have exposed rtPA on the outside of the matrix through chemical conjugation or have nonspecific release when in conditions of pathological thrombosis [39–41]. The target of the thrombolytic carriers is generally the endothelial cells at the vascular wall, and the carriers should increase interaction and adhesion with the target cells. However, direct exposure of most rtPA to the bloodstream can increase the risk of HT through free dissociation and decrease the drug activity through biological inhibitors [42]. Therefore, maximized concealment of rtPA within the carriers may be required for preventing HT.

**Fig. 1:**
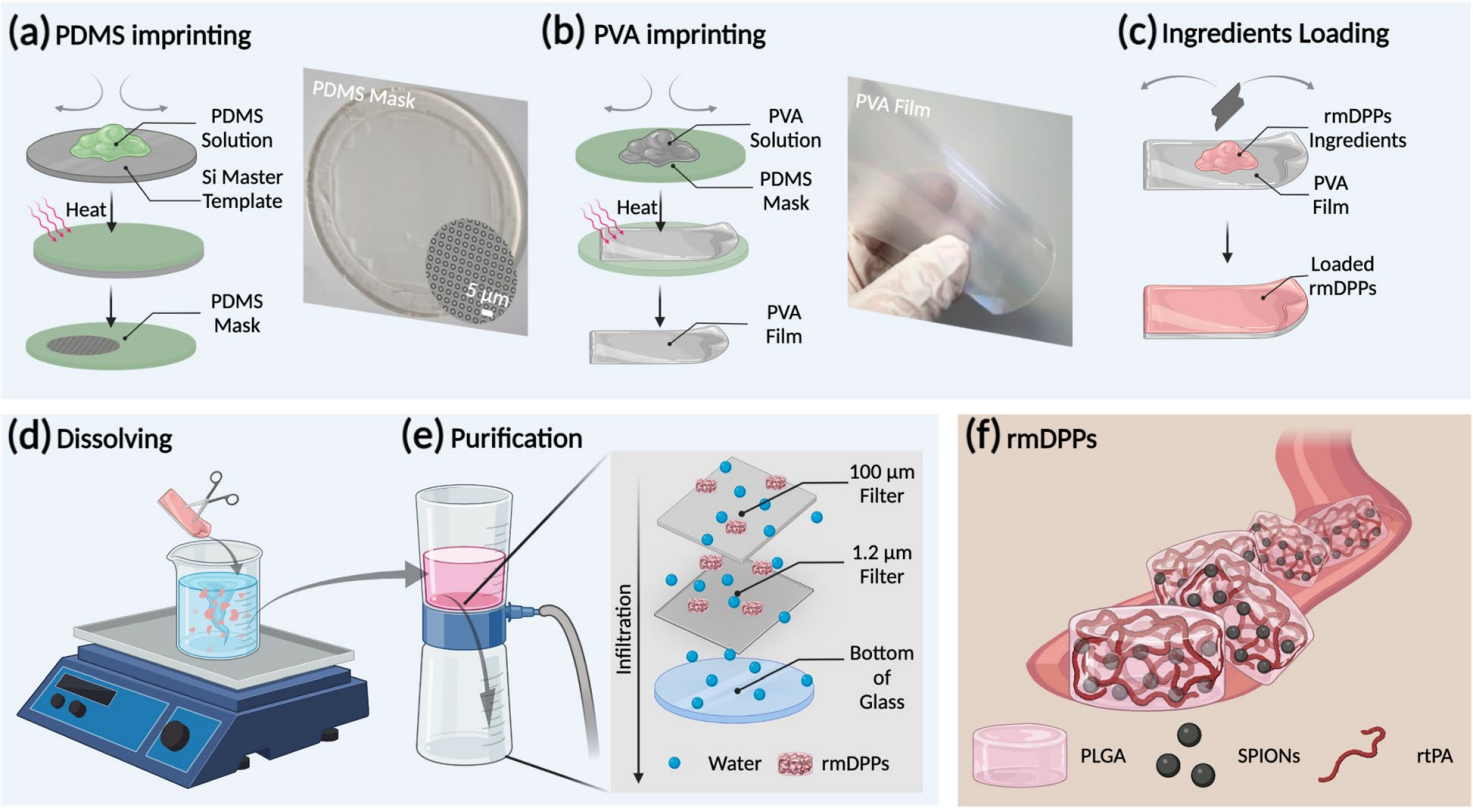
Schematic fabrication procedure of rmDPPs. **(a)** The inverse of the Si master template is acquired by pouring the polydimethylsiloxane (PDMS) solution into the master template. **(b)** A poly(vinyl alcohol (PVA) film with the same structure as the master template is made by pouring PVA solution into the PDMS template. **(c)** Filling the discoidal wells with a polymer paste obtained from poly(lactic-co-glycolic) acid (PLGA), SPIONs, and rtPA. **(d)** The hydrophilic layer of the PVA mask is dissolved in water, and **(e)** flow through membranes with 100 and 1.2 $\upmu$m-sized pores is performed to purify the rmDPPs. **(f)** Schematic illustration of rmDPPs consisting of PLGA, SPIONs, and rtPA in a blood vessel

**Fig. 2:**
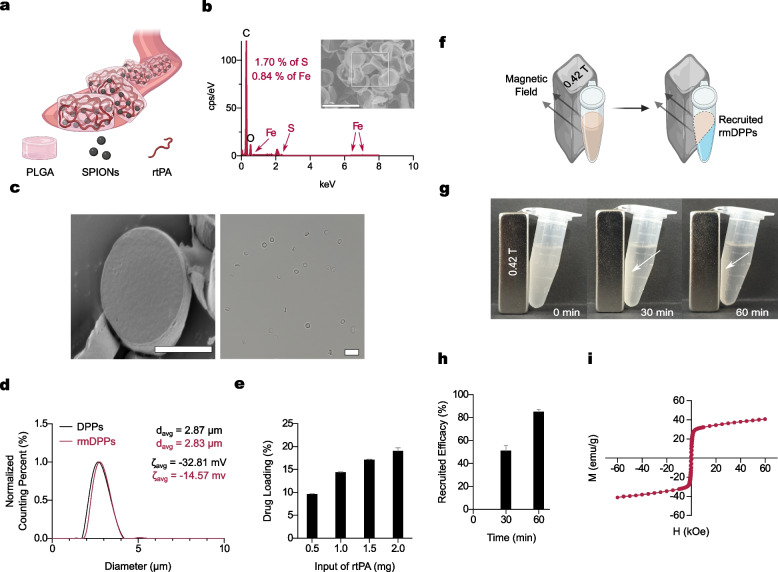
Characterization of rmDPPs. **(a)** Schematic illustration of rmDPPs. **(b)** Detected Fe and S in rmDPPs using energy-dispersive X-ray spectroscopy (EDS) analysis, indicating that SPIONs and rtPA were successfully localized within the PLGA matrix (0.84 wt % for Fe; 1.70 wt % for S, $n=3$). **(c)** Geometric features observed in the scanning electron microscope (SEM) and microscopic images, showing rmDPPs were disc-shaped and uniformly synthesized. **(d)** No dramatic changes in initial size in rmDPPs, indicating loading SPIONs and rtPA into PLGA did not affect the inherent size of DPPs ($n=3$). **(e)** Increased drug loading with the rtPA input ($n=3$). **(f-g)** Schematic illustration and images of recruited rmDPPs depending on reaction time with 30 min intervals, showing that rmDPPs could be localized into the external magnetic field (vertical magnetic field: 0.42 T, $n=3$). **(h-i)** Quantification of recruitment efficacy and presentation of magnetization, indicating that rmDPPs demonstrated superparamagnetic properties ($n=3$)

Consequently, the thrombolytic rtPA carrier should be targeted at the local site and release the drug selectively with an external stimulus. Since this approach enhances the concentration of rtPA at the site of the blood clot by simultaneously exposing the rtPA, which may increase the stability of rtPA and reduce concerns about the additional HT compared to the systemically circulating rtPA. Thus, we reported 3 $\upmu$m discoidal magnetoacoustic particles loaded with rtPA and SPIONs (termed as rmDPPs) and tested the thrombolytic potential in a photothrombotic murine model. We found that the rmDPPs could be targeted in the desired site using magnetic field and burstly release the rtPA under acoustic stimulus. Therefore, we named this approach as magneto-sonothrombolysis applied with magnetic and acoustic treatment for effective thrombolysis. The rmDPPs were successfully synthesized with a biocompatible and biodegradable poly(lactic-co-glycolic) acid (PLGA), rtPA, and SPIONs and were prepared with a top-down fabrication approach (Fig. 1) [43, 44]. A semipermanent polydimethylsiloxane (PDMS) mold was used as the master template to synthesize and shape a disposable poly(vinyl alcohol) (PVA) film (Fig. 1a-b). The water-soluble PVA film contained PLGA, rtPA, and SPIONs, and the rmDPPs were collected through filter purification (Fig. 1c-e). The rmDPPs were approximately 3 $\upmu$m and disc-shaped (Fig. 2c-d and Supplementary Fig. 5); therefore, the design of particles was suitable for stable attachment to the blood clots and lateral movement within the bloodstream (*i.e.*, perpendicular to the direction of flow) [45–48]. Acoustic stimulus controlled the release of rtPA loaded in the rmDPPs (Fig. 4). Direct loading of rtPA into the polymer granted the property of stealth to the rtPA, leading to an underestimated lytic potential *in vitro* compared to the rtPA alone (Fig. 5). However, a thrombolytic potential comparable to that of free rtPA was achieved when magnetic attraction and acoustic stimulus were simultaneously applied to the *in vitro* blood clot. rmDPPs could be targeted *in vivo* with magnetic attraction (Fig. 6). A photothrombotic model was established to investigate the thrombolytic potential of rmDPPs, showing that combining magnetoacoustics with rmDPPs dramatically reduced the infarct aroused from the thrombosis model (Fig. 7). Delayed treatment (*i.e.,* 3 hrs poststroke) was also effective and did not show any HT complications (Figs. 8 and 9). A favorable behavior prognosis was observed in the mice treated with rmDPPs with magnetoacoustic stimuli to show a recovery in locomotor functions comparable to the control group (Fig. 10).

## Materials and Methods

### Materials and Reagents

rtPA was purchased from Boehringer Ingelheim (Ingelheim, Germany). The commercially available rtPA was provided with L-arginine as a stabilizer. Poly (D,L-lactide-co-glycolide) acid (PLGA, lactide:glycolide=50:50, MW=24,800-38,000, 719870), poly (vinyl alcohol) (PVA, 80 % hydrolyzed, MW=9,000-10,000, 360627), dichloromethane (270997), chloroform (C2432), hexane (139386), thrombin from bovine plasma (over 60 NIH units/mg protein, T7326) and N,N’-Dicyclohexylcarbodiimide (DCC, D80002), iron(III) chloride hexahydrate (F2877), 1-Octadecene (O806), oleic acid (364525), RIPA buffer (R0278), phosphatase inhibitors (P0044, P5726), Rose Bengal (95 %, 330000), 3-(2-Pyridyl)-5,6-di(2-furyl)-1,2,4-triazine-5’,5”-disulfonic acid disodium salt (P4272), L-ascorbic acid (A92902), TraceCERT iron standard for ICP (43149), 2,3,5-triphenyl tetrazolium chloride (TTC, over 98 %, T8877), 0.5 % Triton X-100 (T8787), Brij L23 solution (B4184), hemoglobin bovine (H2500), and Drabkin’s reagent (D5941) were provided by Sigma-Aldrich (St. Louis, Mo, USA). Sodium oleate (00057) was purchased from TCI (Tokyo, Japan). N-Hydroxysuccinimide (NHS, 24500), n-Tetracosane (42462), ammonium acetate (A647), 1,1’-Dioctadecyl-3,3,3’,3’-Tetramethylindodicarbocyanine Perchlorate (DiD, D307), and acetic acid (A38) were provided by Thermo Fisher Scientific (Waltham, MA, USA). Protease inhibitors (11836170001) were purchased from Roche (Mannheim, Germany). Polydimethylsiloxane (PDMS, 31-00858-0) 184 silicon Elastomer Kit was purchased from Dow Corning (Midland, Michigan, USA). Isopore Membrane Filter (1.2 $\upmu$m pore size, 47 mm diameter, RTTP04700) and Nylon Net filter (100.0 $\upmu$m pore size, 90 mm diameter, NY1H09000) were provided by Merck Millipore (Burlington, Massachusetts, USA). Customized US transducers were fabricated from Dong I1 Technology (Seoul, Republic of Korea). US transmission gel was provided from SANIPIA (Jeonbuk, Republic of Korea). 532 nm green dot laser module (532nm-dot-LM) was provided from Berlin Laser (Berlin, Germany). Rabbit anti-fibrinogen (MBS315814) was purchased from MyBioSource (San Diego, USA). Mouse anti-beta-actin (SC-47778) was provided from Santa Cruz (Texas, USA).

### Synthesis of Superparamagnetic Iron Oxide Nanoparticles (SPIONs)

The SPIONs were synthesized using a thermal decomposition method presented in a previous study [49]. Briefly, 1.08 g iron(III) chloride and 3.65 g sodium oleate were dissolved in a 28 mL mixture of ethyl alcohol, distilled water (DW), and hexane. The mixture was thermally evaporated at 60 $^{\circ }$C for 4 hrs using a 3-neck flask under constant nitrogen influx to form an iron oleate complex. The mixture was washed with DW three times, and excess remaining hexane was evaporated at 40 $^{\circ }$C for 30 min using a rotary evaporator (SB-1300, Singapore). The collected 1.8 g iron oleate, 0.312 g oleic acid, and 3 g N-tetracosane were dissolved in 9 mL 1-octadecene. The mixture was evaporated at 320 $^{\circ }$C for 1 hr in a 3 neck flask with vigorous stirring to induce the nucleation/growth phase of nanocrystals. The resulting solution was cooled at room temperature, followed by the addition of 15 mL hexane. Synthesized SPIONs were separated from hexane using a magnetic field, and the SPION-containing solution was washed three times with acetone. The images and X-ray diffraction (XRD) pattern of SPIONs nanocrystals were obtained using transmission electron microscopy (TEM, JEM-F200, Japan) and XRD machine (D8 Advance, Bruker, USA) to characterize the crystals, respectively.

### Synthesis of rmDPPs

The synthesis procedure of rmDPPs is presented in Fig. 1. rmDPPs were synthesized through the top-down fabrication, following our previous method [43, 44]. The procedure for the top-down fabrication could be classified into 5 steps: fabrication of PDMS mask, PVA film imprinting, components loading into the PVA film, dissolving, and purification of particles. First, the 3 $\upmu$m-sized Si master template was prepared with a lithography technique. The mixture of PDMS solution and reducing agents (10:1, *w/w* %) was poured into the master template (Fig. 1a). The mixture was dried out at 60 $^{\circ }$C for 4 hrs to polymerize the resulting solution. The polymerized PDMS layer was carefully removed from the master template and was used as a semipermanent counterpart template for disposable PVA films which have the same structures as those of the Si master template. Second, the PVA film was fabricated (Fig. 1b). 48 mg of PVA powder were dissolved in 800 mL of DW, followed by stirring at 120 $^{\circ }$C for 2 hrs. The liquified PVA solution was poured into the fabricated PDMS layer, and the polymerized PVA layer was collected. Third, the loading components were adequately prepared to load those into the PVA film (Fig. 1c). The loading components consisted of 70 mg of PLGA, 500 $\upmu$g of SPIONs, and 1 mg of rtPA. All materials were dissolved in the 250 $\upmu$L of co-solvent consisting of hexane, DCM, and chloroform. The mixture dissolved in the co-solvent was evenly loaded in the disposable PVA film using a razor blade. Fourth, the rmDPPs could be released from the PVA film through a water-abundant environment (Fig. 1d). The film was dissolved in DW for 3 hrs. Finally, the purified rmDPPs could be collected by membrane filtration with pore sizes of 100 $\upmu$m and 1.2 $\upmu$m (Fig. 1e). A schematic of the rmDPPs is shown in Fig. 1f. The rmDPPs contained PLGA as a polymeric matrix, SPIONs as a magnetic targeting moiety, and rtPA as a thrombolytic agent.

### Characterization of rmDPPs

Dynamic light scattering (DLS) was used to assess the size and zeta potential of the particles. A Multisizer (Mastersizer 3000, UK) was utilized for accurate size measurement of the rmDPPs since the shape of the particles was not homogeneously spherical. The zeta potential evaluation was performed using a Zetasizer (Nano ZS90, UK). All particles were dispersed in DW at a 0.2 mg/mL concentration for size and zeta potential measurements.

Morphological information of rmDPPs was assessed using scanning electron microscopy (SEM, Inspect F50, USA) and bright-field microscopy (Eclipse Ti 2, Japan). 10 $\upmu$L of rmDPPs (1 mg/mL) was diluted with 390 $\upmu$L of DW, and the mixture was suspended in a confocal dish for microscopic imaging.

Element distribution was analyzed by energy-dispersive X-ray spectroscopy (EDS) to validate whether both rtPA and SPIONs were successfully localized within the polymeric structure. EDS analysis was performed simultaneously with SEM imaging.

The colloidal stability of rmDPPs was assessed through size variation. The test was performed in both phosphate-buffered saline (PBS) fetal bovine serum (FBS) (30%, *v/v*). 10 $\upmu$L of particles (1 mg/mL) was diluted with 20 mL of ISOTON II diluent and exposed to 37 $^{\circ }$C in a heat blocker (JSOF-100, South Korea). Stability evaluation was performed once per day on days 1-4 and on day 7.

Drug loading (%) was quantified using spectroscopic measurements. It was necessary for rtPA to be conjugated with a fluorescent substance to quantify the drug loading spectroscopically; therefore, rtPA was covalently bonded with Rhodamine B (RhoB-rtPA). A more detailed procedure for chemical bonding is described in Supplementary Fig. 1. rmDPPs were prepared with different masses of RhoB-rtPA (0, 0.5, 1, 1.5, and 2 mg). The structure of the rmDPPs was disrupted by adding 1 mL of DMSO to allow leakage of the loaded RhoB-rtPA from the PLGA matrix. The standard curve was regressed using RhoB-rtPA (0, 0.0625, 0.125, 0.25, 0.5, and 1 mg/mL). Absorbance of rmDPPs was measured through a Multimode Reader (HTX Multi-Mode Reader, USA) at 450 nm.

Recruiting efficacy with magnetic attraction was evaluated using a 0.42 T magnet. rmDPPs (1 mg/mL) were exposed to the magnet for 60 min, and recruited masses near the magnetic field were assessed at 30 min intervals. Attracted rmDPPs were collected through the withdrawal of supernatant DW and freeze-dried to quantify the mass. Recruiting efficacy toward the 0.42 T magnet was calculated as:

$$\begin{aligned} Recruiting\; Efficacy=m_{collected}/m_{input} \times 100 \end{aligned}$$

where the *m* is the mass.

The magnetic properties of rmDPPs were evaluated at 5 $^{\circ }$C using a vibrating sample magnetometer (MPMS3 SQUID magnetometer, UK). The hysteresis loop determined the magnetization of particles at 300 K in the alternating magnetic field between -60 and 60 kOe.

### Acoustic Systems

An unfocused transducer (UT) and focused transducer (FT) were employed for *in vitro* and *in vivo* magneto-sonothrombolysis, respectively. Both UT and FT were customized to have the same resonance frequency at 1.5 MHz. The acoustic setup consisted of a function generator (AFG3022B, USA), linear power amplifier (210L, USA), transducer, and oscilloscope (DPO4104, USA). Mechanical pressure from the transducer was delivered into a calibrated needle-type hydrophone (HNR-0500, USA) as sampled electrical signals, which were further processed to calculate acoustic distribution and intensity (AI). Acoustic parameters, such as fundamental frequency (FF), tone-burst duration (TBD), pulse repetition frequency (PRF), and duty cycles (DC), were regulated using the function generator.

The hydrophone was moved in 3-axis spatial volume using a robotic platform (Bislides, US) to precisely describe the acoustic features of transducers under acoustic stimulus. The movement of the platform was controlled through MATLAB. Acoustic characterization was performed in degassed water. The UT and FT were each characterized at a focal depth and geometrical focus: 100 mm and 13 mm, respectively. Acoustic distribution in both UT and FT was examined in the lateral, vertical, and axial directions. Depending on the various electrical inputs from the function generator, the AI of both UT and FT transducers was characterized with the 1.5 MHz FF, 100 $\upmu$s TBD, 1 kHz PRF, and 10 % DC parameters. The AI was described as spatial-peak temporal-average intensity (I$_{SPTA}$) in this configuration. For AI calculation, we used a density of water of 1028 kg/m$^3$and a speed of sound in the water of 1515 m/s [50]. The I$_{SPTA}$ in both transducers was fixed at approximately 12.34 W/cm$^2$ in both *in vitro* and *in vivo* magneto-sonothrombolysis experiments. The I$_{SPTA}$ was defined as follows:

$$\begin{aligned} I_i=p_i^2/\rho c \end{aligned}$$

$$\begin{aligned} PII=\int _{t_1}^{t_2} I_i^2 dt \end{aligned}$$

$$\begin{aligned} I_{SPTA}=PII\times PRF \end{aligned}$$

where p is the pressure, $\rho$ is a density of water, and c is the speed of sound.

### Acoustic Responsiveness of rmDPPs

Two approaches for testing the acoustic responsiveness of rmDPPs were validated. First, 200 $\upmu$L of RhoB-rtPA-loaded rmDPPs solution (1 mg/mL) was prepared inside a confocal dish. The confocal dish was mounted on a homemade housing system and treated by sonication. The acoustic stimulus was maintained for 0, 1, and 5 min. The reaction solution was centrifuged at 4,000 RPM for 90 s to separate the damaged rmDPPs and leaked rtPA. The supernatant was collected to acquire the optical density (OD). The OD was regressed to the standard curve using 1 mg of RhoB-rtPA-loaded rmDPPs, followed by subtracting the control value from each OD. Approximately 143.8 $\upmu$g of RhoB-rtPA was loaded in the 1 mg RhoB-rtPA-loaded rmDPPs. A biodegradability assay was performed to directly compare US-mediated rtPA leakage with rtPA release owing to PLGA hydrolysis.

Second, an enzymatic activity assay was performed with the US stimulus. This assay can confirm the ability of rtPA to activate plasminogen to plasmin, releasing a yellow para-nitroaniline (pNA) chromophore in solution. Overall enzymatic assay was performed with the diluted solution following the protocol of manufacture. The rmDPPs were synthesized with different loading amounts of rtPA: 0, 0.1, 0.5, 1, and 2 mg. The activity of native rtPA was also evaluated to validate whether the acoustic stimulus toward the rmDPPs could release the cargoes more. The particles treated with/without US were reacted through an enzymatic kit. The investigations were maintained for 120 min with 30 min intervals, and enzymatic activity was quantified by measuring the OD at 405 nm. The result was presented as accumulative rtPA enzymatic activity relative to the control (*i.e.,* mixture consisting of normal saline and assay reagents) at each investigated time.

### Assessment of the Magnetoacoustic Potential of rmDPPs in Fibrinolytic Gel Model

A slightly-modified fibrinolytic gel model was developed to examine the magnetoacoustic potential of rmDPPs for targeted and tunable rtPA release [42]. An agarose solution contained thrombus-related components and zymogen, and the color of the agarose turned white when it was fully polymerized. When rtPA was added to the gel, the polymerized fibrin content in the agarose gel was expected to be lysed and transparent. Briefly, to create the gel, 20 mL of agarose solution (0.5%) was stirred on a magnetic stirrer with 2 mL of fibrinogen (10 mg/mL), 10 $\upmu$L of thrombin (1 kU/mL), and 300 $\upmu$L of plasminogen solution (2.5 mg/mL). The resulting solution (1 mL) was stored in a vertical channel at 37 $^{\circ }$C for 1.5 hrs to fully polymerize. The treatment dose of the rmDPPs was adjusted to be equal to that of the rtPA (*i.e.,* 10 $\upmu$g). Either 100 $\upmu$L of normal saline (*i.e.*, control), rtPA, or rmDPPs was added to the top of the vertical channel. To evaluate the magnetic recruitment potential of the rmDPPs, a 0.42 T neodymium magnet was placed at the bottom of the channel. The channels subjected to US stimulus were sonicated for 10 min. The polymerized gels were carefully withdrawn from the channel after 24 hrs to quantify the magnetoacoustic potential of rmDPPs. Quantification was performed by using NIH ImageJ software with binary image processing.

### Assessment of the Magneto-sonothrombolysis Potential of rmDPPs Using In Vitro Blood Clots

All animal experiments were approved by the Institutional Animal Care and Use Committee of the Korea Institute of Science and Technology (KIST-2021-06-073) and performed with appropriate recommendations. Blood was obtained from Sprague-Dawley rats weighing 280 g by the heart puncture method to fabricate *in vitro* blood clots. Briefly, the rat was anesthetized with a mixture of oxygen and isoflurane, followed by a 5 mL syringe with 21 G into the left ventricle. Then, 20 U of thrombin (1 kU/mL) and 70 $\upmu$L of collected blood were reacted in the Eppendorf tube to obtain highly retracted blood clots in a 37 $^{\circ }$C incubator. The blood clots were carefully removed from the tube after 30 min and placed on the confocal dish to test the magnetoacoustic potential of the rmDPPs. The blood clots were treated with 10 $\upmu$L of normal saline (*i.e.*, control), rtPA, or rmDPPs equal to 10 $\upmu$g of rtPA. For magneto-sonothrombolysis, the 0.42 T magnet was placed on the bottom of confocal dish for 20 min, followed by sonicating the dish for 10 min. Lysis of blood clots (*i.e.,* the dissolution of hemoglobin) was quantified by spectroscopy at 415 nm after 60 min.

### In Vivo Fluorescence Imaging (IVIS)

To monitor the targeting potential of rmDPPs *in vivo*, near-infrared carbocyanine fluorescent (NIRF) DiD-loaded rmDPPs (2 mg/200 $\upmu$L) were injected into 8-week-old BALB/C nude mice through intravenous (IV) systemic injection ($n=6$). To fabricate DiD-loaded rmDPPs, approximately 300 $\upmu$g of DiD was loaded together with the solution containing PLGA, SPIONs, and rtPA. 200 $\upmu$L of normal saline without any particles was administered to a control group. The 0.42 T neodymium magnet was simply attached onto the skull surface in the right hemisphere with bio-silicon to allow biased direction in targeting, followed by injecting the particles systemically. All NIRF images were acquired 20 min postinjection using the IVIS Lumina Series III (PerkinElmer, USA) at 10 min intervals. The NIRF intensities were quantified as radiant efficacy using Living Image software.

### Assessment of the Magnetoacoustic Potential of rmDPPs Using an In Vivo Photothrombotic Model

A platelet-rich photothrombotic model was established for the *in vivo* model with slight modification [51]. Briefly, 10-week-old BALB/C male mice (approximately 25-28 g) were anesthetized through intraperitoneal injection (IP) with a cocktail of Zoletil 50 and xylazine (3:1, *v/v*), depending on the weight of the mouse. A mixture of Rose Bengal (15 mg/mL) and thrombin (80 U/kg) was injected into the IV route. To induce acute ischemic stroke, the mice were transcranially irradiated by a 532 nm green dot laser onto the area covering motor 1 and broad somatosensory cortex for 10 min. We prepared two *in vivo* experiments. First, we compared the thrombolytic potential of rmDPPs with that of rtPA through treatment at 20 min poststroke (*i.e.*, within the guideline time). Second, we compared the potential of rmDPPs with that of rtPA at 3 hrs poststroke (*i.e.*, relatively over the guideline time). The control mice were injected with 200 $\upmu$L of normal saline in both experiments. For the treated mice, 200 $\upmu$L of treatment solution was administered through IV tail injection at a dose of 10 mg/kg for rtPA or 2 mg/mouse for rmDPPs. Both rtPA and rmDPPs were dissolved in normal saline. For testing the magneto-sonothrombolysis of particles, the exposure times of the magnetic and acoustic stimuli were 20 and 10 min, respectively. After finishing the photothrombotic surgery, the mice were moved to a fresh cage without light and were monitored for acute toxicity symptoms. The mice were humanely sacrificed to collect the whole brain 1 day poststroke. The brains were sectioned at 1 mm intervals, and TTC solution was used to stain the infarcts. The infarct was quantified using NIH ImageJ software.

### Immunoblotting of Fibrinogen Content in the Ipsilateral Hemisphere

Immunoblotting for fibrinogen was performed to examine whether fibrinogen was decreased in the ipsilateral hemisphere depending on the magneto-sonothrombolysis treatment with rmDPPs. Briefly, the brain was divided into the contralateral and ipsilateral hemispheres, and each hemisphere was homogenized with the mixture consisting of RIPA buffer, phosphate inhibitors, and protease inhibitors using a hand-held homogenizer (D1000, USA) on ice. A Bradford assay was employed to quantify the total protein concentration in each hemisphere. The target protein was visualized by HRP-reactive chemiluminescence reagents and quantified using a blotting imaging system (iBright 750, USA). The antibodies used for immunoblotting were rabbit anti-fibrinogen and mouse anti-$\beta$-actin.

### Quantification of Intracerebral HT

Intracerebral HT was quantified using a cyanmethemoglobin assay as previously described elsewhere [52]. First, the mice were induced by photothrombosis and then injected with the appropriate substances for experimental purpose. At 1 day poststroke, the mice were perfused through transcardiac puncture with 100 mL of phosphate-buffered saline, and then the brains were collected. The ipsilateral brains were homogenized in 500 $\upmu$L of phosphate-buffered saline with 0.5 % Triton X-100. The lysates were centrifuged at 13,000 $\times$ g for 30 min. The collected supernatant of 200 $\upmu$L was mixed with 800 $\upmu$L of Drabkin’s reagent and stored at room temperature for 15 min. The resulting solution was placed in a 96-well plate, and the OD at 540 nm was measured to quantify intracerebral hemorrhage. The calibration curve was regressed using the cyanmethemoglobin standard solution including 180 mg/mL hemoglobin. The standard solution was prepared in Drabkin’s reagent following the manufacturer’s instructions. The measured OD was compared with the calibration curve, and the intracerebral HT was presented in units of mg/mL.

### Safety Issues

We evaluated the histological safety of acoustic stimulus and hematological toxicity of rmDPPs. A total of 4 mice were prepared for hematoxylin and eosin (H &E) staining to validate histological safety of acoustic stimulation. Acoustic parameters were the same as those used for *in vivo* magneto-sonothrombolysis. The collected brains were fixed in 4 % paraformaldehyde for 1 day at room temperature, followed by coronal sectioning using a cryostat (CM1950, Leica Microsystems). Five sections were obtained at intervals of 200 $\upmu$m from the center point of the acoustic stimulus, and the thickness of each section was 20 $\upmu$m. All procedures for cryosectioning and H &E staining were performed with a standard protocol. A total of 6 mice were involved to test hematological toxicity of rmDPPs ($n=3$ for control; $n=3$ for rmDPPs). The control and rmDPPs groups were treated with 200 $\upmu$L of normal saline and rmDPPs (2 mg/200$\upmu$L), respectively. The fresh blood was acquired using heart punctuation in the left ventricle and stored in a EDTA-treated Vacutainer. The representative complete blood count (CBC) analysis was performed using a blood cell counter (XN-V, Japan).

### Behavior Testing

Behavioral testing was performed to examine the recovery of locomotor function after rmDPPs treatment. We examined the locomotor function using a rotarod (99-0061, Laiyue). All mice were trained and recorded for their behavioral features prior to the photothrombotic stroke ($n=4$ per group). The initial speed of the motor was 4 rad/s. The speed was increased to the 40 rad/s within 3 min. The maximum speed of the rotarod was plateaued at 3 min. All testing were maintained for 5 min. We investigated the latency to fall down from the rotarod wheel to present the locomotor function.

### Statistical Analysis

All plotting and statistical analyses of data were performed using GraphPad Prism software. All data are presented as the mean ± standard deviation (SD). The Shapiro-Wilk test confirmed the normality of the data, and significant differences between the data were compared using unpaired t test and one-way analysis of variance (ANOVA) with *p* values (*<0.05, **<0.01, ***<0.001) indicating statistical significance.

## Results

### Synthesis and Characterization of rmDPPs

The SPIONs were observed to be a spherical and 10.70 nm in size by the TEM images ($n=2053$, Supplementary Fig. 2), showing that the overall nucleation and growth phases in the nanocrystals were successful. The typical pattern of SPIONs presented peaks at 2$\theta$ at 30.1, 35.4, 42.9, 56.9, and 62.3°in the XRD, assigning to diffraction of the (220), (311), (400), (511), and (440) planes, respectively (Supplementary Fig. 3). A considerable portion of Fe (0.84 wt %) and S (1.70 wt %) was measured in rmDPPs by EDS analysis (Fig. 2b and Supplementary Fig. 4), which is attributed to the disulfide bonds in rtPA and fundamental composition of SPIONs. SEM and microscopic images showed that the particles were disc-shaped and uniformly synthesized (Fig. 2c and Supplementary Fig. 5); the rmDPPs had an averaged pitch of 973.8 nm ($n=6$) and diameter of 2.67 $\upmu$m ($n=4$). Naive particles (*i.e.,* DPPs that did not contain any substance) were additionally fabricated to compare their physicochemical properties with those of rmDPPs. According to Multisizer measurements, the average sizes of DPPs and rmDPPs were 2.87 and 2.83 $\upmu$m, respectively (Fig. 2d, $n=3$). The results of size measurement indicated that the loading procedure with rtPA and SPIONs into PLGA could not dramatically affect the inherent size of the DPPs. The electrostatic charges of DPPs and rmDPPs were -32.81 ± 0.30 and -14.57 ± 0.14, respectively, according to zeta potential measurements (Fig. 2d, $n=3$). The slightly shifted zeta potential in rmDPPs was related to the positively charged amino acid in the additive L-arginine that stabilized rtPA. Drug loading (%) was investigated according to the rtPA input. Drug loading values of 9.64 ± 0.10, 14.38 ± 0.106, 17.12 ± 0.14, and 19.05 ± 0.70 % were measured in rmDPPs with 0.5, 1, 1.5, and 2 mg of rtPA input, respectively (Fig. 2e, $n=3$).

The colloidal stability of rmDPPs was evaluated at 37 $^{\circ }$C in the PBS and FBS and continued for 7 days (Table 1). The stability in size was maintained in both DPPs and rmDPPs for 2 days in the PBS condition, although a slight decrease in both sizes was observed after the 2nd day. A 17.07 and 20.14 % decrease in size were measured in the DPPs and rmDPPs, respectively, after 2 days compared to the initial size of each (2.87 ± 0.14 to 2.38 ± 0.10 $\upmu$m for DPPs; 2.83 ± 0.06 to 2.26 ± 0.02 $\upmu$m for rmDPPs). A decrease in size of 50.17 and 51.59 % were estimated in the DPPs and rmDPPs, respectively, after 7 days compared to each original size (2.87 ± 0.14 to 1.43 ± 0.11 $\upmu$m for DPPs; 2.83 ± 0.06 to 1.37 ± 0.19 $\upmu$m for rmDPPs). More rapid decrease tendency in the size was observed in the FBS condition. Approximately 14.80 and 16.53 % decrease in the colloidal stability were assessed in the DPPs and rmDPPs, respectively, after 1 day compared to the original size (2.95 ± 0.09 to 2.51 ± 0.09 $\upmu$m for DPPs; 2.83 ± 0.23 to 2.36 ± 0.06 $\upmu$m for rmDPPs). The 56.39 and 59.90 % decrease in the DPPs and rmDPPs were evaluated in the FBS condition at post 7 days (2.95 ± 0.19 to 1.29 ± 0.06 $\upmu$m for DPPs; 2.83 ± 0.23 to 1.13 ± 0.09 $\upmu$m for rmDPPs). The decrease in size stability in both DPPs and rmDPPs might be related to the hydrolysis of the PLGA matrix in a suspension, leading to a similar variation in size stability.

**Table 1:** Colloidal stability of rmDPPs in the PBS and FBS. Standard deviation for SD; Polydisperse index for PDI

PBS							FBS (30 %, *v/v*)					
Time (days)	Size of DPPs ($\upmu$m)	SD	PDI	Size of rmDPPs ($\upmu$m)	SD	PDI	Size of DPPs ($\upmu$m)	SD	PDI	Size of rmDPPs ($\upmu$m)	SD	PDI
0	2.87	0.14	0.11	2.83	0.06	0.04	2.95	0.09	0.15	2.83	0.23	0.05
1	2.82	0.03	0.12	2.81	0.18	0.04	2.51	0.09	0.18	2.36	0.06	0.14
2	2.38	0.10	0.13	2.26	0.02	0.13	2.26	0.06	0.15	2.17	0.08	0.14
3	2.27	0.22	0.19	2.22	0.40	0.14	2.07	0.02	0.12	1.92	0.09	0.15
4	2.12	0.14	0.20	1.82	0.21	0.17	1.62	0.21	0.14	1.48	0.24	0.16
7	1.55	0.40	0.18	1.42	0.19	0.19	1.29	0.06	0.18	1.13	0.09	0.19

The recruitment efficacy and magnetization of rmDPPs were also investigated (Fig. 2f-i). The rmDPPs in suspension were recruited toward the 0.42 T external magnet (Fig. 2g). Recruitment efficiencies of 51.00 ± 4.58 % and 85.00 ± 2.00 % were measured at 30 and 60 min, respectively, relative to 0 min (Fig. 2h, $n=3$). Magnetization saturation was observed at 40.8 and 41.2 emu/g without any remanence or coercivity in the loop behavior (Fig. 2i). These results show that the particles could be attracted toward an external magnetic field since the loaded SPIONs inherently exhibited superparamagnetic properties.

### Acoustic System Characterization

**Fig. 3:**
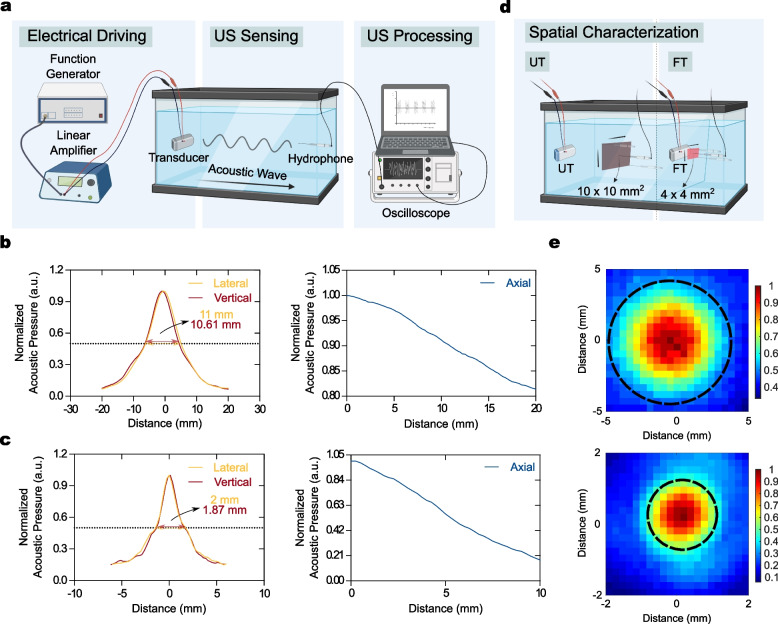
Characterization of the acoustic system. **(a)** Schematic characterization of the full width at half maximum (FWHM). The acoustic pressure was driven from the linear amplifier connected with a function generator, and then the pressure was measured by hydrophone to convert the mechanical output into electrical signals. **(b-c)** FWHM of UT and FT, showing that a larger FWHM was detected in the UT than in the FT. **(d)** Schematic procedure of spatial characterization (Investigated size: 10 $\times$ 10 mm$^2$ for UT; 4 $\times$ 4 mm$^2$ for FT). **(e)** Evaluation of the focal diameter at -6 dB, indicating that 8.57 and 1.91 mm were measured in UT and FT, respectively

Supplementary Fig. 6 shows both *in vitro* and *in vivo* sonothrombolysis transducers and each housing system. An *in vitro* housing system was fabricated to equip the UT and confocal dish at the bottom and top sides of the system, respectively (Supplementary Fig. 6a). The acoustic housing system for *in vivo* sonothrombolysis was manufactured using a 3D printing technique to equip the FT. The *in vivo* housing was 10 mm in diameter and 13 mm tall and could be filled with transmission gel to radiate the acoustic beam (Supplementary Fig. 6b). Both transducers were characterized to acquire the full width at half maximum (FWHM), spatial beam distribution, and AI. Figure 3a shows a schematic of the FWHM characterization. The FWHM in UT in the lateral and vertical directions was measured as 11 and 10.61 mm, respectively (Fig. 3b, left). It should be noted that the glass with a diameter of 13 mm is acoustically transparent; therefore, the smaller FWHM of UT which was smaller than the diameter of glass indicated that the overall energy could be radiated into the dish. The FWHM in the FT in the lateral and vertical directions was 2 and 1.87 mm, respectively (Fig. 3c, left). Likewise the UT, because the beam thickness of the 532 nm laser used for the photothrombotic model was a 2.8 mm with the manufacturer’s specifications, the energy could be stimulated into the desired ipsilateral hemisphere. Characterizations of both transducers in the axial direction showed a decline in acoustic pressure along with the distance (Fig. 3b and c, right), showing that the characterization was well performed at either the geometrical or focused plane. Figure 3d presents the schematic procedure for acquiring the spatial beam distribution using the UT and FT. The distributions of the UT and FT were measured in 10 $\times$ 10 mm$^2$ and 4 $\times$ 4 mm$^2$ squares, respectively. Considering the visualization of the beam distribution, approximately 8.57 and 1.91 mm of focal diameter were measured at -6 dB in the UT and FT, respectively (Fig. 3e, dash line). A spatial-peak temporal-average intensity, referred to as I$_{SPTA}$, was a 12.34 W/cm$^2$.

### rtPA Release from rmDPPs by US Stimulus

**Fig. 4:**
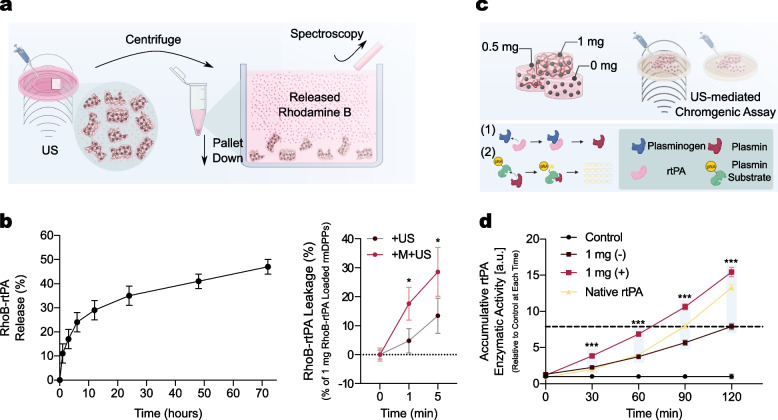
US stimulus-mediated release of rtPA from rmDPPs. **(a)** Schematic procedure of rtPA release depending on the acoustic stimulus. **(b)** Biodegradability assay for quantification of rtPA release due to PLGA hydrolysis **(left)** and quantification of rtPA leakage due to the +US and +M+US **(right)**, confirming the burst release of rtPA was feasible through the magnetoacoustic approach in the rmDPPs. **(c)** Schematic mechanism of the measurement of enzymatic activity. **(d)** Quantification of the enzymatic activity depending on the acoustic stimulus. Plasmin substrates contained the chromophore pNA and released pNA when they reacted with plasmin, demonstrating the enzymatic activity though the colorimetric method was successful. 1 mg of rtPA was presented as the optimal drug loading in the polymeric structure, showing the highest cumulative enzymatic activity among the test groups (all $n=5$)

The rtPA should be released as soon as possible from the carriers to treat time-sensitive thrombotic diseases. First, spectroscopic analysis was performed to quantify the amount of rtPA leakage from rmDPPs. After using US to stimulate the solution containing rmDPPs, all damaged or undamaged particles were spun down through centrifugation. The quantification of leaked rtPA was possible by measuring the relative OD of the supernatant (Fig. 4a). Acoustic-mediated rtPA leakage was compared to the result in the biodegradability assay depending on the PLGA hydrolysis (Fig. 4b). The release of rtPA due to the hydrolysis of PLGA matrix was quantified as 11 % at 1 hr, 17 % at 3 hrs, 24 % at 6 hrs, 29 % at 12 hrs, and 35 % at 24 hrs. Approximately 41 % and 47 % of rtPA release was measured after 2 and 3 days, respectively (Fig. 4b, left). The release of RhoB-rtPA gradually increased with the sonication time (Fig. 4b, right). Approximately 6.90 and 19.40 $\upmu$g of RhoB-rtPA was released from the carriers within 1 and 5 min by acoustic stimulus alone, respectively. When the magnetoacoustic approach was applied to the rmDPPs, approximately 17.60 and 28.60 $\upmu$g of rtPA was released from the carriers. This result demonstrates that the simultaneous acoustic and magnetic stimuli could accelerate the release of cargoes from the discoidal structure. Also, it should be noted that approximately 1 hr was required to release 14.70 $\upmu$g of RhoB-rtPA from the carriers with natural hydrolysis. The level of release could be fulfilled within 5 and 1 min with acoustic alone and magnetoacoustic stimuli, respectively.

rtPA is a typical protein-based thrombolytic agent. Notably, the top-down fabrication procedure was performed in organic solvent. Therefore, it was necessary to check whether the enzymatic activity of rtPA was maintained after the synthesis and US stimulus could enhance the leakage of rtPA from the carriers. Figure 4c presents a schematic of the enzymatic activity assay of rtPA. When plasmin was reacted with a plasmin substrate containing pNA, the chromogenic substrate could be released in a reaction solution. The enzymatic activity of rtPA in the rmDPPs could be either maintained or increased with acoustic stimulus (Fig. 4d). Additionally, approximately two times higher accumulated profile was observed in the rmDPPs loaded with 1 mg of rtPA in comparison with the same rmDPPs without US stimulus. There was no significant difference in the rmDPPs loaded with 0.5 mg of rtPA regardless of acoustic stimulus. For the enzymatic activity in 0.1 and 2 mg of rtPA-loaded rmDPPs, unstable activities were measured along with the reaction time (Supplementary Fig. 7). These results indicate that the loading of rtPA into the polymer did not disturb the original activity of the drug, and even the activity could be accelerated by US stimulus.

### In Vitro Fibrinolytic and Blood Clot Lysis Using rmDPPs

The fibrinolytic potential of the gel treated with rtPA was investigated in a dose-dependent manner (Supplementary Fig. 8). Although more rapid lysis was observed in the gel with the incremental rtPA dose, 10 $\upmu$g of rtPA was sufficient to lyse half of the gel within 24 hrs. Therefore, the height of the fibrinolytic gel was reduced by half, and the optimized rtPA dose was adjusted to 10 $\upmu$g.

**Fig. 5:**
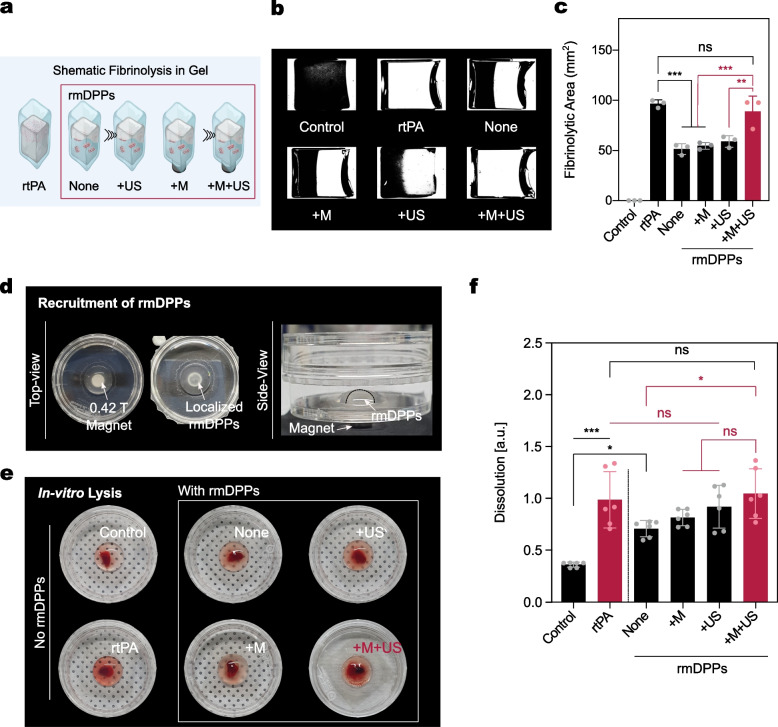
Assessment of *in vitro* fibrinolytic and thrombolytic potential of rmDPPs. **(a)** Schematic fibrinolytic gel model. **(b)** Binary images processing of lysed gel, showing that rtPA induced whole gel lysis within the observed time, while both None, +M, and +US groups did not lyse the gel effectively. Slightly different lytic patterns were observed in the +M+US group, presenting nonlytic areas in the one-side bottom or in the center. A more detailed description of lytic patterns is presented in Supplementary Fig. 9. **(c)** Quantification of fibrinolytic gel area after 24 hrs ($n=3$). **(d)** Localized rmDPPs using the 0.42 T magnet in the dish, showing that the particles were seen in the site where the magnet was located (top view). Separated phase between water and rmDPPs in suspension (side view). **(e)** Images of rmDPPs-mediated *in vitro* blood clot lysis with various external stimuli. **(f)** Quantification of blood clot dissolution ($n=6$). Group information: rmDPPs without any external stimulus for the None group; rmDPPs with sole magnetic attraction for the +M group; rmDPPs with sole acoustic stimulus for the +US group; rmDPPs with the simultaneous stimuli for the +M+US group

Figure 5a presents a schematic fibrinolytic gel model. Additionally, binary image processing was performed to visualize the boundary between the polymerized gel and the lysed area (Fig. 5b and Supplementary Fig. 9). The lysed area was quantified using these binary-processed images (Fig. 5c). The control group showed no lytic area. Whole lysis of the polymerized gel was observed in the rtPA-treated group within 24 hrs, whereas treatment with rmDPPs did not accelerate the degradation of the gel compared to that with rtPA. In particular, 96.60 % of the gel was lysed within 24 hrs when treated with rtPA, although 51.34 % of the fibrinolytic potential was measured in the rmDPPs group without external treatment (*i.e.,* the None group), which was approximately 46.85 % lower than that of rtPA (p<0.001). This might be related to the limited exposure of rtPA from the polymeric matrix in rmDPPs. The 54.69 and 59.01 % gel degradation was observed with magnetic and acoustic stimulus alone, respectively (*i.e.,* +M and +US, respectively). This indicates that the sole external stimulus with rmDPPs could not significantly improve the lysis of the gel. However, the +M+US group showed an approximately 88.97 % of the gel lysis. The lytic potential in the +M+US group was approximately 1.73-, 1.63-, and 1.51-fold higher compared to that in the None, +M, and +US groups, respectively (p<0.001 for None and +M; $p=0.003$ for +US). The transparent area between the rtPA and +M+US groups was not significantly different ($p=0.79$). Interestingly, although the lytic pattern of rtPA was multidirectional, that of the +M+US group was shown to be either unidirectional or concreted at the center of the magnetic field (Supplementary Fig. 9). These results demonstrate that rmDPPs could be advantageous for promoting rtPA stealth by approximately half or more compared to rtPA in a fibrinolytic gel model.

The aspirated blood from the rat heart was mixed with thrombin to reproducibly fabricate blood clots, followed by incubation in the conical tube. Such conical blood clot lysis models have been widely utilized to test the lytic potential of various thrombolytic agents [53–55]. The added thrombin produces a more retracted and stable blood clot [56]. Although overall the blood clots were highly controlled in their shape through the matured thrombosis reaction with thrombin, some blood clots showed an unexpected lytic potential with normal saline (Supplementary Fig. 10a). Therefore, only highly structured blood clots were chosen to test the blood clot lysis potential using magnetoacoustic rmDPPs. Before we evaluated the *in vitro* magneto-sonothrombolytic potential of rmDPPs, the potential of rtPA dose-dependent blood clot lysis was first investigated (Supplementary Fig. 10b). It was revealed that 10 $\upmu$g of rtPA sufficiently induced lysis; therefore, we fixed the optimal dose of the drug to 10 $\upmu$g. Hereafter, all investigations related to *in vitro* thrombolysis utilized 10 $\upmu$g of rtPA or 10 $\upmu$g of rtPA-containing rmDPPs.

Due to the magnetic properties of rmDPPs, rmDPPs in suspension were effectively recruited in the 0.42 T magnetic field (Fig. 5d). The OD in the control was 0.62 ± 0.04, while that of rtPA was 1.70 ± 0.47 (Fig. 5e-f). Approximately 2.73-fold greater lysis was observed in the rtPA-treated blood clots compared to the control (p<0.001). When rmDPPs were applied to the blood clots, the OD was presented as 1.22 ± 0.13, showing 1.96- fold greater lysis compared to the control ($p=0.02$). There were no significant differences between the None and +M and +US groups ($p=0.90$ for +M; $p=0.33$ for +US), suggesting that external stimulus alone with particles could not enhance the blood clot lysis. The OD in the +M+US group was 1.80 ± 0.41, showing the highest thrombolytic value among the test groups. This was an approximately 47.63 % increase in lytic potential when compared to the None group ($p=0.03$). However, there was no statistically significant difference in the lytic potential between the +M+US and rtPA, indicating that rmDPPs with magnetoacoustic approach could induce a similar lytic potential to rtPA.

### In Vivo Targeting Potency of rmDPPs

**Fig. 6:**
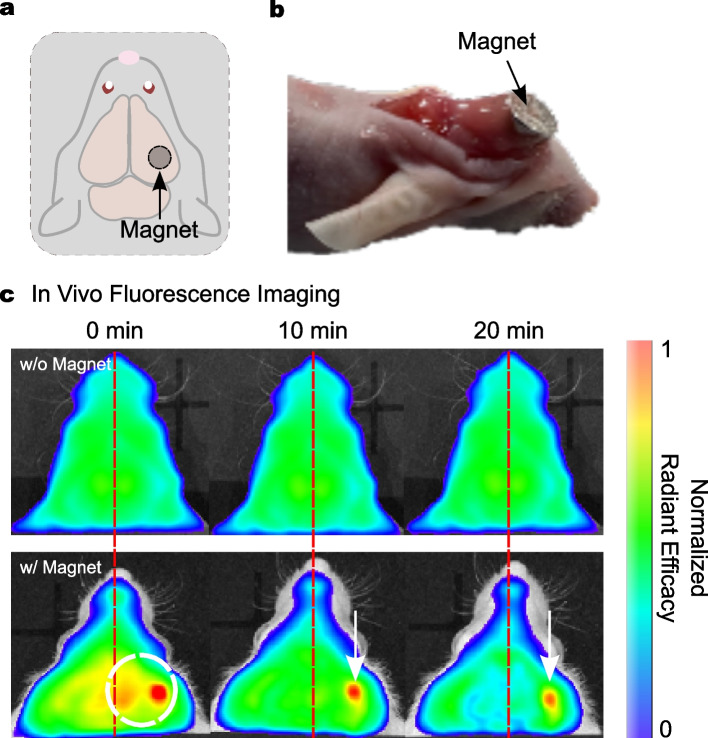
Representative NIRF images obtained using the *in vivo* fluorescence imaging system. **(a-b)** Schematic illustration and representative image of a magnet-attached mouse in the right hemisphere. **(c)** Typical NIRF images for mice treated with the rmDPPs w/o and w/ magnetic attraction, showing that no localized NIRF intensities along with hemispheres were observed in the w/o magnet group, while the biased intensities were presented in the right hemisphere in w/ group. White circle for magnet-placed location; White arrow for the localized rmDPPs zone

It was necessary to evaluate the targeting potential of rmDPPs with magnetic attraction in the presence of complex physiological barriers *in vivo*. We assessed whether rmDPPs were targeted in brain blood vessels using *in vivo* fluorescence imaging (Fig. 6). NIRF spectra with/without magnetic guidance (*i.e.*, w/ and w/o) were acquired and presented as normalized radiant efficacy. First, we investigated the basic pharmacokinetics of the freely circulating rmDPPs. The circulating particles mainly accumulated in the lung (Supplementary Fig. 11a). Since the relatively high NIRF intensities in the lungs, it was hard to distinguish whether the particles were feasibly targeted in the neurovessels with the magnetic field. A small portion of signals was detected near the brain, however, it could not precisely demonstrate whether the particles were localized in the vessels (Supplementary Fig. 11b). Therefore, we changed the field of view closer to the brain. Figure 6a and b show a schematic illustration for biased magnetic placement and typical image of a magnet-attached mouse onto the skull surface, respectively. The bio-silicon was used to maintain the position of the magnet on the skull during the recruitment and could be easily detached. No concreted NIRF spectra in the w/o group were shown in both hemispheres. In contrast, the mice selectively given magnetic attraction in the right hemisphere presented strong NIRF intensities in that hemisphere (Fig. 6c). Consequently, using the external magnetic field, rmDPPs could be brought to the vicinity of the cerebral blood vessels at the desired position within 20 min.

### In Vivo Magneto-sonothrombolysis Using rmDPPs

The magneto-sonothrombolytic potential of rmDPPs was assessed in a photothrombotic model. In advance, the dose-dependent effect of rtPA on the infarct was first evaluated to optimize the appropriate rtPA dose for *in vivo* (Supplementary Fig. 12). The 75 $\upmu$g rtPA treatment did not reduce the infarct compared to the 0 $\upmu$g rtPA treatment ($p=0.95$) and showed inconsistent lytic potential (11.55 ± 4.99 mm$^3$, $n=3$). The 150 and 300 $\upmu$g rtPA treatments reduced the infarct, showing volumes of 5.06 ± 3.37 ($n=5$) and 3.96 ± 2.35 mm$^3$ ($n=4$), respectively. This was a reduction in volume of approximately 60.06 and 75.35 % of alleviation in the volume compared to the 0 $\upmu$g rtPA treatment ($p=0.003$ for 150 $\upmu$g; $p=0.002$ for 300 $\upmu$g). Although there was no significant difference in the infarct between the 150 and 300 $\upmu$g of rtPA ($p=0.95$), we designated the administration dose of the drug as 300 $\upmu$g, following the previous reference [51].

**Fig. 7:**
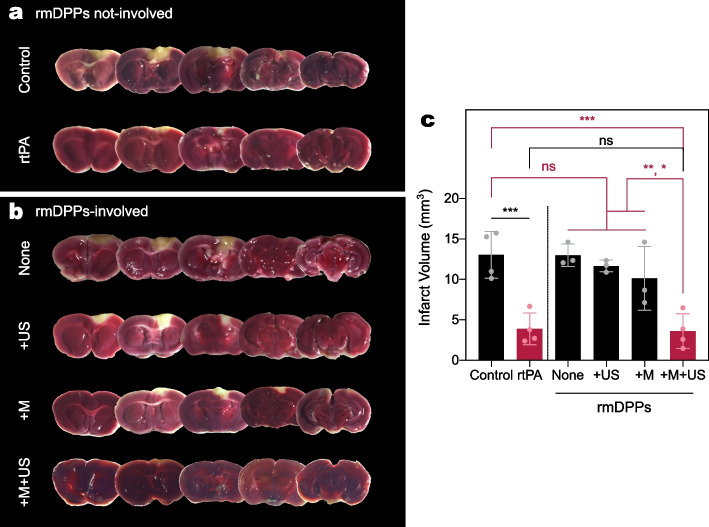
Evaluation of the *in vivo* magneto-sonothrombolytic potential of rmDPPs. **(a-b)** Typical TTC-stained brain slices after the respective treatments. **(c)** Quantification of the infarct using the TTC-stained brain slices ($n=4$ for the control, rtPA, and +M+US groups; $n=3$ for None, +US, and +M groups), showing that only successful recanalization in cerebral vessels was achieved through simultaneous magnetic and acoustic stimuli with rmDPPs. Group information: rmDPPs without any external stimulus for None group; rmDPPs with sole magnetic attraction for +M group; rmDPPs with sole acoustic stimulus for +US group; rmDPPs with the simultaneous stimuli for +M+US group

Figure 7a and b show typical TTC staining results. All quantifications of infarcts were based on the TTC-stained images (Fig. 7c). The infarct in the control was estimated as 13.03 mm$^3$ ($n=4$). Similar to the previous rtPA pilot study, a reduction in infarct volume of approximately 70.10 % was observed in rtPA-treated mice (p<0.001). However, no infarct volume reduction was observed in the None group (p>0.99). Approximately 1.00-fold infarct was assessed compared to the control, indicating that circulating rmDPPs *in vivo* could not exhibit lytic potential toward blood clots. The infarcts in the +M and +US groups were measured as 10.14 and 11.66 mm$^3$, respectively. However, both the +M and +US groups were not significantly different from the control ($p=0.63$ for +M; $p=0.97$ for +US), indicating that a single external stimulus with rmDPPs could not increase the thrombolytic potency.

The infarct in the +M+US group was assessed as 3.61 mm$^3$, showing the highest reduction potential among the test groups. This was an approximately 72.31, 64.43, and 69.07 % reduction in the infarct volume compared to the control, +M, and +US groups, respectively (p<0.001 for control; $p=0.03$ for +M; $p=0.006$ for +US). However, there was no significant difference in the infarcts between the rtPA and the +M+US groups. Although the rmDPPs caused a reduction in the infarcts through combinatorial treatment with magnetic and acoustic stimuli, dramatic improvement beyond the potential or rtPA alone was not observed due to the limited exposure of rtPA in the matrix, which was consistent with our *in vitro* lysis results.

Since the administration of rtPA at the 20 min poststroke is within the recommended time, we designed a photothrombotic model in which mice were injected with either rtPA or particles beyond this time. Currently, the rtPA should be injected into the thrombotic patients within 3-4.5 hrs of stroke symptom onset. The therapeutic efficacy of rtPA beyond this time is presented in Supplementary Fig. 13. Unlike the previous pilot study that delivered rtPA at 20 min poststroke, all doses were insufficient to reduce the infarct (all p>0.05). There were no significant differences between the treatments with 75, 150, and 300 $\upmu$g of rtPA. Therefore, the late administration of rtPA did not function in the photothrombotic model, which is consistent with clinical recommendations.

**Fig. 8:**
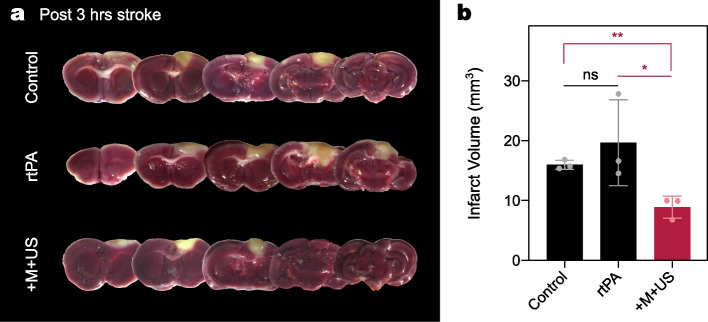
Assessment of magneto-sonothrombolytic potential in control, rtPA, and +M+US group for the infarct in delayed treatment (post 3 hrs stroke). **(a)** Representative TTC-stained brain slices for the mice (control, rtPA, and +M+US group). **(b)** Quantification of the infarct, indicating that delayed treatment of rtPA did not work well in the infarct improvement and only +M+US group induced reduction in the volume ($n=3$)

Figure 8a shows the representative TTC staining results from the mice treated with the respective reagents at 3 hrs poststroke. The quantification of the infarct was also performed using TTC-stained brain slices (Fig. 8b). The infarct in the control was measured as 15.98 mm$^3$. There was no significant difference between control and rtPA-treated mice ($p=0.44$), which was consistent with the fact that the late injection of rtPA was not effective in the model [51]. However, the infarct in the +M+US group was confirmed to be 8.91 mm$^3$, approximately 44.25 % lower than that in the control ($p=0.004$). Therefore, the magneto-sonothrombolytic potential of rmDPPs in the ischemic stroke could effectively recanalize the obstructed superficial vessels even after 3 hrs poststroke, whereas bolus rtPA injection could not function well.

### Assessment of Immunoblotting and Intracerebral Hemorrhage

**Fig. 9:**
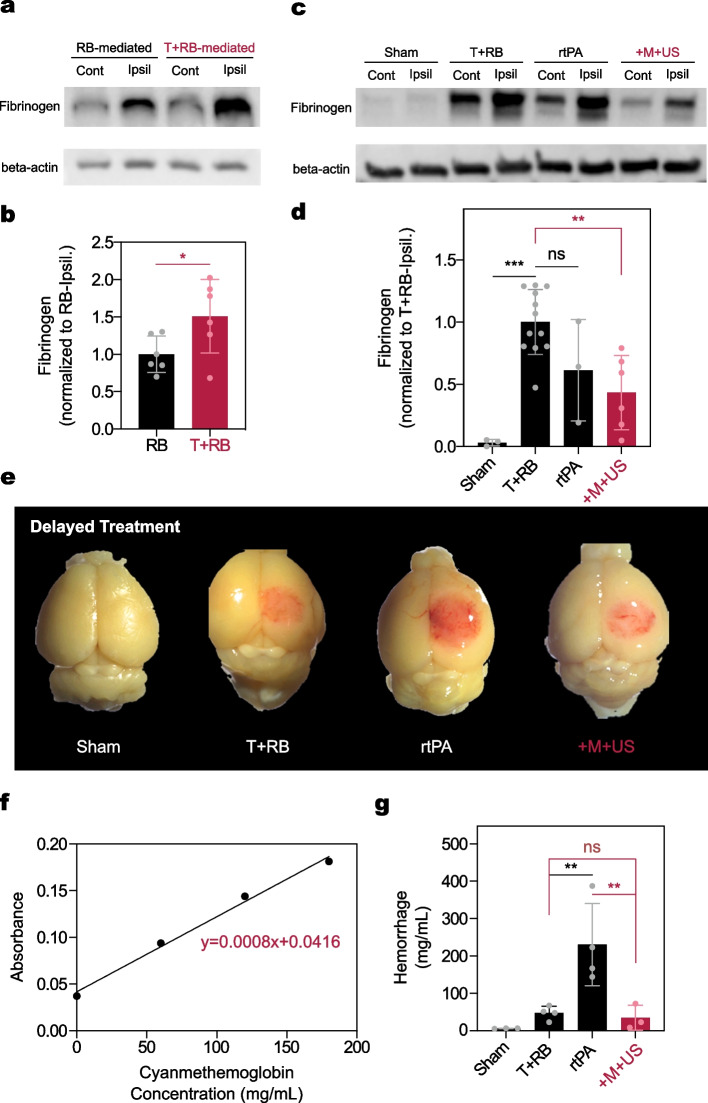
Immunoblotting of fibrinogen in the ipsilateral hemisphere and quantification of intracerebral hemorrhage. $\beta$-actin was used for immunoblotting housekeeping genes (normal saline treatment for sham; Rose Bengal (RB) with thrombin treatment for T+RB; rtPA treatment for rtPA; rmDPPs with magneto-sonothrombolytic approach for +M+US group). **(a-b)** Typical immunoblotting and quantification of fibrinogen in the RB and T+RB groups, showing stable platelet deposits were observed in the ipsilateral hemisphere compared with RB alone (*p* = 0.047). **(c-d)** Typical immunoblotting and quantification of fibrinogen expression in the sham, T+RB, rtPA, and +M+US groups, showing that rtPA treatment showed not only an unstable fibrinolytic potential (standard deviation: 0.507, $n=5$), but also no significant difference was detected when compared to the T+RB (T+RB for $n=12$; rtPA for $n=3$; $p=0.16$). There was no significant difference between the rtPA and +M+US groups ($p=0.80$). **(e)** Typical image of intracerebral HT after delayed treatment with either drug or particles with a magneto-sonothrombolytic approach. **(f-g)** Regressed cyanmethemoglobin standard curve and quantification of intracerebral HT, showing delayed treatment of rtPA induced the HT ($n=4$), whereas the +M+US group did not present any HT ($n=3$)

The photothrombotic model has been generally established using the Rose Bengal (RB). Recent observation demonstrated that thrombin added with RB (T+RB) could produce the blood clot with an appropriate fibrin ratio and platelets [51]. We first compared the immunoblotting results of RB and T+RB to validate that fibrinogen was well deposited in the ipsilateral hemisphere in our condition. Figure 9a and b shows the typical immunoblotting result and quantification of fibrinogen expression with RB and T+RB, respectively. Approximately 1.63-fold greater fibrinogen was deposited in the T+RB-treated ipsilateral hemispheres than in the RB-treated ones ($p=0.047$) (Fig. 9b). Normal saline treatment with photoactivation did not produce fibrinogen deposits in the hemisphere, showing only approximately 5.03 % of T+RB in the fibrinogen deposit in the T+RB condition (Fig. 9c). This might be a naturally expressed level of fibrinogen in the hemisphere. rtPA induced highly unstable degradation potential in the blotting and showed the highest standard deviation in the level of fibrinogen among all groups (standard deviation: 0.51, Fig. 9d). Therefore, the expression of fibrinogen deposits with rtPA was almost the same as that of T+RB ($p=0.16$). However, the +M+US showed deposits reduced by as much as 43.14 % for T+RB ($p=0.003$), suggesting that elevated fibrinolysis potential could be accomplished by treatment with the magneto-sonothrombolytic approach using rmDPPs through degradation of the deposits.

Figure 9e shows typical images of brains administered with the different treatments after 3 hrs poststroke. All brains were damaged by the photothrombotic activation, except for the brain acquired from the sham group. Additionally, the rose-colored substance was observed in each ipsilateral hemisphere, which might be attributed to be the leakage of RB *via* the blood-brain barrier (BBB) disruption. Although additional HT in the ipsilateral hemisphere was detected in the delayed rtPA-treated group, the +M+US group showed low HT in the hemisphere. Intracerebral HT was quantified using a cyanmethemoglobin assay. The cyanmethemoglobin standard curve was regressed to y=0.0008x+0.0416 (Fig. 9f). Consistent with the previous images, the HT in the T+RB and rtPA-treated groups was assessed as 47.16 and 230.18 mg/mL, respectively (Fig. 9g). The HT in the rtPA-treated group was approximately 4.88-fold greater than that in the T+RB group ($p=0.009$). The +M+US condition showed 34.38 mg/mL of HT, which was the lowest hemorrhage value among the test groups. Approximately 6.69-fold more HT was quantified in late rtPA-treated mice than in the +M+US group ($p=0.01$). There was no significant difference between the T+RB and +M+US treated groups (p>0.99). In summary, the magneto-sonothrombolytic potential of rmDPPs did not lead to additional HT in the intracerebral region, even after late treatment.

### Behavior Testing

**Fig. 10:**
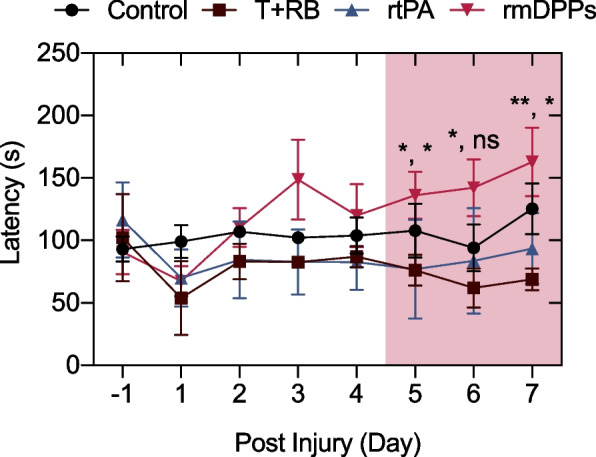
Recovery of locomotor function. The reproducible enhancement in the locomotor function was observed in the rmDPPs with magnetoacoustic stimuli, showing comparable capability to the control. There were significant differences between rmDPPs group and rtPA and T+RB groups at 5 days post-injury ($p=0.045$ for T+RB and $p=0.048$ for rtPA at 5 days post-injury; $p=0.015$ for T+RB at 6 days post-injury; $p=0.002$ for T+RB and $p=0.015$ for rtPA at 7 days post-injury)

Figure 10 presents the representative results of the rotarod behavior testing. The initial training onto the rotarod was well established among the mice (Fig. 10). Approximately 50-70 s of latency was examined after the acute photothrombotic stroke in T+RB, rtPA, and rmDPPs group, whereas the latency in control was highly maintained over the 100 s. The parameter in the T+RB group tended to be restored when continuing the rotarod test and thus was similar to the value of the rtPA group after 2 days post-injury. However, in the rmDPPs group, the latency was increased with the progression of the rotarod test, showing highly similar values to the control group. The favorable prognosis in the locomotor functions was well recognized after 5 days post-injury, compared to the T+RB and rtPA ($p=0.045$ for T+RB and $p=0.048$ for rtPA at 5 days post-injury; $p=0.015$ for T+RB at 6 days post-injury; $p=0.002$ for T+RB and $p=0.015$ for rtPA at 7 days post-injury). This indicates that the rmDPPs could prompt the locomotor function in the photothrombotic model through effective recanalization in the occluded neurovessels and induce favorable prognosis.

## Discussion

This study proposes a method to relieve infarcts without side effects such as intracerebral HT in the ischemic photothrombotic model while dissolving blood clots using magnetoacoustic rmDPPs that limit the inherent thrombolytic function of rtPA as much as possible.

We demonstrated that the SPIONs and rtPA were successfully loaded within the polymeric structure (Fig. 2b and Supplementary Fig. 4). Since the PLGA used as the polymeric structure is a synthetic polymer consisting of C, H, and O in chemical, the detected both Fe and S in the EDS analysis could be originated from the SPIONs and rtPA, respectively. Comprehensively, the rmDPPs could involve the overall materials by simply loading them into the template paste and could be targeting drug carriers. Since the nanoscale particles generally required significant time to be accumulated in the target area in the bloodstream, the rmDPPs encapsulating a relatively large amount of SPIONs in polymeric structure might be superior to recruitment.

We checked that the rtPA release from the rmDPPs could be feasible using acoustic stimulus within a relatively short time compared to the natural hydrolysis of the PLGA (Fig. 4b). Especially, it should be noted that the acoustic stimulus could release the approximately 20 $\upmu$g of rtPA from the carriers within 5 min, whereas the rmDPPs could release the 15 $\upmu$g of rtPA for 1 hr depending on the hydrolysis. It is noted that the magnetoacoustic stimuli with rmDPPs could also accelerate the release of rtPA from the carriers compared to that of acoustic alone. This magnetoacoustically release of rtPA in the rmDPPs is well conditioned in our experimental design. Furthermore, the functional activity of native rtPA was well maintained regardless of before/after synthesis, and the activity of rmDPPs without acoustic stimulus was underestimated compared to that of native rtPA (Fig. 4d). However, the activity started to increase with acoustic stimulus, which corresponded to the leakage of rtPA within the carriers. Regarding the release/leakage mechanism of rtPA in the rmDPPs, this might be possible through physical destruction by acoustic stimulus involving either cracks or defects on the surface of the particles, which is supported by a previous study [36–38]. The consideration of safety in acoustic stimulus was necessary since polymeric particles might need a higher acoustic stimulus to release the cargo than the conventional microbubbles widely used in the sonothrombolysis field. Our acoustic stimulus could not induce any severe damage in the sonicated region, such as intracerebral hemorrhage, through the H &E staining (Supplementary Fig. 14). Furthermore, the hematological parameters acquired using rmDPPs were not significantly different from the control, showing the application of particles *in vivo* might be safe (Table 2). Consequently, the rmDPPs could be synthesized in a fixed space with a limited polymer to be easily disrupted by acoustic stimulus (Supplementary Fig. 15).

**Table 2:** Hematological safety of rmDPPs

Parameter	Control	rmDPPs
RBC (M/$\upmu$L)	9.83 ± 0.41	10.27 ± 0.10
HGB (g/dL)	14.25 ± 0.49	15.50 ± 0.17
HCT (%)	47.15 ± 1.20	49.40 ± 0.52
MCV (fL)	47.95 ± 0.78	48.13 ± 0.29
MCH (pg)	14.50 ± 0.14	15.10 ± 0.20
MCHC (g/dL)	30.20 ± 0.29	31.40 ± 0.35
RDW (%)	17.15 ± 1.06	18.87 ±0.12
MPV (fL)	7.15 ± 0.35	8.07 ± 0.06
PLT (K/$\upmu$L)	759.00 ± 1.41	709.67 ± 114.21
WBC (K/$\upmu$L)	5.79 ± 2.43	4.61 ± 0.78
NEU (%)	18.70 ± 0.28	18.70 ± 3.16
LYM (%)	77.70 ± 2.40	78.43 ± 2.89
MONO (%)	1.00 ± 0.42	1.77 ± 0.85
EOS (%)	2.10 ± 1.98	0.93 ± 0.81
BASO (%)	0.50 ± 0.28	0.17 ± 0.15

Conventionally, relevant investigations in acoustic-assisted drug delivery have employed the microbubbles consisting of gases and lipids [25, 57]. Although the gas-phase microbubbles could lyse the blood clots by dispersing cavitational energy into the surrounding bloodstream, this might provide the surrounding tissues with high rarefactional pressures or locally increased temperature [58]. Therefore, recent studies have attempted to develop more stable and homogeneous particles using amphiphilic polymeric substances, showing polymeric micelles and hybrid-based microbubbles [59]. The other previous works successfully encapsulated the rtPA into the PLGA polymer [60, 61]. These polymeric particles were synthesized by an emulsion technique as a bottom-up method. The self-assembly structure in the PLGA nanoparticles could provide high loading efficacy of rtPA. The self-assembly nanoparticles could be generally synthesized as a spherical structure. However, modification of shape and chemical composition between the individual particles might be hard. Considering that the required time for half amount of loading rtPA to be released was approximately 3 days from rmDPPs (Fig. 4b), the relatively release profile of rtPA within 1 day might be short in the other polymeric rtPA carriers. Furthermore, in the aspect of the targeting drug carriers, the rmDPPs might be more advantageous in the targeting capability compared to the nanoscale particles since the rmDPPs volumetrically contained the clustered SPIONs with the polymeric matrix. The high tendency to be recruited toward static magnetic field in rmDPPs is correlated to the high yield in the rtPA release in the precise blood vessels, which might lead to favorable prognosis in the ischemic stroke treatment.

In the aspect of the design of thrombolytic carriers, the interaction between nanoparticles and blood clots in the bloodstream might be complicated in comparison with the rmDPPs. The particles with over 500 nm size tend to marginate toward the vascular wall due to the gravitational forces [62, 63]. It was reported that the margination tendency in blood vessels could be increased in the particles with size from 500 nm to 5 $\upmu$m, whereas the particles larger than 5 $\upmu$m showed the inverse tendency in circulation [64, 65]. It should be noted that the size of rmDPPs was approximately 3 $\upmu$m. Therefore, the rmDPPs might not require much systemic circulation time to interact with target endothelial cells compared to the nanoparticles [66]. Additionally, since disc-shaped particles showed to move laterally and thus increased the chances of interacting with blood clots which are generally elongated from endothelial cells [45–48]. However, one of the crucial considerations for developing thrombolytic carriers is to understand the correlation between the release profile of rtPA and the HT. Although the previous works reported the enhanced thrombolytic potential through the hydrolysis of polymeric structure to release the rtPA, the relationship between their release profile and HT complications was not fully investigated. Therefore, the selective rtPA release in the ischemic vessels might be needed to develop an safe thrombolytic drug delivery system and reduce the additional HT problems.

The ability to localize drug carriers at the lesion location prior to selective drug release is also important consideration for successful thrombolysis. Especially, the thrombolytic drug carriers could penetrate within the blood clot structure to achieve high thrombolytic efficacy. Several reports adopted the fibrinolytic gel model or showed penetrated drug carriers as real images [42, 67]. Our results in the fibrinolytic gels presented that the rmDPPs without any stimulus could result in 50 % lower lysis compared to the native rtPA (Fig. 5b-c). The comparable fibrinolytic potential of rtPA could be acquired in the rmDPPs with simultaneous magnetic and acoustic stimuli. Comprehensively, the loading procedure that the rtPA was localized in the polymeric structures through the top-down fabrication could limit the exposure of rtPA. Interestingly, the interpretation that the whole rtPA was limited to exposure within the polymer might be difficult. This might be related to the naturally porous structure of the PLGA matrix [68, 69] and the hydrophilic property of loaded rtPA, which leads to exposure of the hydrophilic functional groups of the rtPA to reaction suspension during the synthesis stage. In short, a particular portion of rtPA could be successfully lyophilized by being trapped in the polymeric matrix, while exposure to other fragments of trapped rtPA is inevitable. However, the close packing of rtPA within the matrix could physically prevent the dissociation of rtPA and thus decrease the level of additional HT [42]. Furthermore, these targeting potential of rmDPPs was also evaluated *in vivo* (Fig. 6). Consequently, although circulating rmDPPs induced no significant differences in the NIRF intensities between the hemispheres, biased rmDPPs using magnetic attraction created a localized zone of particles in a specific right hemisphere. According to our previous study, the 3 $\upmu$m discoidal particles synthesized by the top-down fabrication were distributed in order of lung, liver, and spleen within 2 hrs postinjection [43]. Therefore, the targeting potential of rmDPPs to specific microvessels through a magnetic field with just 20 min is not only a favorable feature for time-sensitive thrombosis, but also suggests that the pharmacokinetics of particles can be intentionally altered. Additionally, no mice involved in NIRF imaging showed any immediate lethality, indicating that the targeting of specific neurovessels was free from safety issues.

Although targeting of either thrombolytic agents or drug carriers could expect a high quality of prognosis in thrombotic patients, the relevant clinical trials have not been sufficiently performed. Regarding our best knowledge, the various magnetic particles challenged to the clinical phase have been utilized for the contrast agents in magnetic resonance imaging. Since the intensities of the magnetic field applied to the human might be required stronger to target magnetic particles in the desired area, more sophisticated translational investigations should be needed for developing these drug delivery carriers. A recent investigation using *in vitro* human artery model demonstrated that the SPIONs having large size and reducing repulsive force from the surface coating could achieve high accumulative efficacy at the same magnetic gradient [70]. However, the toxicity of endothelial cells due to the high accumulation might limit the use in the clinical field. Since matrices of rmDPPs contained the SPIONs, a direct comparison with previous studies that utilized the SPIONs themselves in the aspect of toxicity may be somewhat complicated. However, the microscale particles could require relatively less magnetic intensity and time to target these particles into the ischemic vessels due to their size. Since it is hard for the discoidal particles to be leaked out of the blood vessels to affect the cellular uptake until these are fully degraded, the potentiated cytotoxicity of the SPIONs might not be problematic.

Generally, the RB-mediated photothrombosis model induces a poor lytic tendency toward rtPA. Recent observations have indicated that the co-injection of RB with a typical coagulation factor, thrombin, can prompt thrombosis that is reactive to rtPA administration [51]. This simple and effective approach was also established in our *in vivo* investigations (Figs. 7-9). Furthermore, although unstable degradation of the fibrinogen was observed in the brain of mice treated with rtPA, our rmDPPs could reduce the fibrinogen level in the ipsilateral hemisphere through magnetoacoustic strategy (Fig. 9c-d). These immunoblotting results in the rmDPPs could also demonstrate that the reduction in the infarcts could be fulfilled through dissolving blood clots since the level of fibrinogen was reduced. Overall results *in vivo* were highly consistent with the previous work [51].

Although the infusion of rtPA at 20 min poststroke was effective in relieving the infarcts, the circulating rmDPPs could not be functional to reduce the infarcts, presenting a similar level of infarcts to the control (Fig. 7). This might be related to the relative stealth function of rtPA in the rmDPPs, as previously discussed. The comparable effect to relieve the infarcts in the rmDPPs could only be acquired using simultaneous magnetic and acoustic stimuli (Fig. 7b-c). Consistently, these results suggest that effective thrombolysis using the rmDPPs could require the targeted/controlled release of rtPA. The notable strength of rmDPPs was demonstrated in the delayed treatment of the drug. Although the magnetoacoustic strategy with the rmDPPs could reduce the infarcts through the thrombolysis without any HT issue, the risk of HT was increased in the native rtPA (Figs. 8 and 9e-g). Furthermore, the locomotor function could be restored in the rmDPPs-treated mice at the level of control even if the photothrombotic model was established (Fig. 10). The representative mechanisms of intracerebral HT after the ischemia are classified into proteolysis, oxygen stress due to the generation of reactive oxygen species, and leukocyte infiltration [71]. The delayed administration of rtPA could generally lead to deposits of the drug in the brain parenchyma and upregulate the matrix metalloproteinase family [72, 73], which could induce the opening of BBB and additional HT. Since the rtPA is the typical proteolytic molecule widely expressed in either neurons or neurovessels, the delayed treatment of rmDPPs could not positively affect these mechanisms through the magnetoacoustic approach. Therefore, the interpretation that the rmDPPs with the simultaneous stimuli could improve the damaged blood flow and lysis of the blood clots expressed in both the larger ischemic and peri-infarcts cores might be more appropriate. Several relevant works demonstrated the method to reduce the HT arose from the rtPA by simply injecting neuroprotective materials. For example, the materials are 17$\beta$-estradiol and cilostazol [74, 75]. The results of these previous studies slightly differ from ours in that they focused on the role of the protective mechanism operating at the cellular level, rather than the controlled exposure and delivery of rtPA in a thrombotic model. The introduced materials could protect the BBB consisting of the endothelial cells by activating the relevant signaling pathways. Additionally, it should be noted that these studies utilized a hypoxia model, such as a MACO, rather than using the thrombotic model. Therefore, the reduction in the HT might be explained by protecting BBB integrity from the damages due to the rtPA.

## Conclusions

In summary, we proposed that rmDPPs with magnetoacoustic features are effective thrombolytic carriers for thrombolysis without any HT complication. The particles can be directed to the thrombosis-induced site, and the on-demand release of the drug can be done by the acoustic stimulus. The release mechanism is thought to be the physical disruption of the particles. In contrast to conventional spherical nanoparticles, the pharmacokinetic properties of rmDPPs are advantageous for traveling through the bloodstream. The direct loading of rtPA into the PLGA matrix limited the therapeutic potential of the drug. As a limitation of this work, the rmDPPs with magnetoacoustic stimuli could not enhance the blood clot lysis potential compared to those without stimulation for *in vitro* examination. Although the infarcts were reduced in the rmDPPs with magnetoacoustic stimuli, there was no significant enhancement over the rtPA. Interestingly, the HT complications were observed in the delayed treatment of the drug, whereas the rmDPPs with magnetoacoustic stimuli did not induce any severe HT. However, the fact that comparable thrombolytic potential of rtPA could be fulfilled in the rmDPPs using the multiple stimuli might still limit the usage of the system in the conventional thrombotic patients. Nonetheless, these discoidal drug delivery systems encapsulated the rtPA into the matrix is apparently appropriate in delayed thrombotic patients to provide successful thrombolysis and reduction of side effects. Future research is expected to develop and examine drug carriers containing various drugs, such as anti-coagulant and anti-platelet drugs, to enhance drug potential.

## Supplementary Information

**Additional file 1:** Supporting Information.

## Data Availability

All data is available upon request to the corresponding author.

## Abbreviations

rtPA: Recombinant tissue plasminogen activator
SPIONs: Superparamagnetic iron oxide nanoparticles
rmDPPs: 3 μm discoidal polymeric particles loaded with rtPA and SPIONs
PLGA: Poly(lactic-co-glycolic) acid
PDMS: Polydimethylsiloxane
PVA: Poly(vinyl alcohol)
HT: Hemorrhagic transformation
IV: Intravenous
US: Ultrasound
DW: Distilled water
DLS: Dynamic light scattering
SEM: Scanning electron microscopy
TEM: Transmission electron microscopy
RhoB-rtPA: Rhodamine-B bonded with rtPA
UT and FT: Unfocused/focused transducer
FF, TBD, PRF, and DC: Fundamental frequency, tone-burst duration, pulse repetition frequency, and duty cycle
OD: optical density
FWHM: Full width at half maximum
+M: with only magnetic stimulus
+US: with only acoustic stimulus
+M+US: with simultaneous magnetic and acoustic stimuli
RB: Rose Bengal
T+: Additive thrombin
