# Supplementary material for: Targeted thrombolysis by magnetoacoustic particles in photothrombotic stroke model
Source: Biomater Res. 2022 Oct 22;26:58. doi: 10.1186/s40824-022-00298-y (PMC9587564; doi:10.1186/s40824-022-00298-y)
Supplement: Supplementary file 1 — Additional file 1: Supporting Information. [file 40824_2022_298_MOESM1_ESM.docx]

RESEARCH ARTICLE

**Supporting Information**

Targeted Thrombolysis by Magnetoacoustic Particles in Photothrombotic Stroke Model

Wonseok Choi^^[[1]](#footnote-1)^,2^, Hyeyoun Cho^2^, Gahee Kim^2^, Inchan Youn^1,3,4^, Jaehong Key^2^[[2]](#footnote-2)^^ and Sungmin Han^1,3*^

^^

**Supplementary Figure 1.** Fluorescence images of rmDPPs loaded with 1 mg of RhoB-rtPA, indicating that the loading procedure of rtPA within the PLGA matrix was successful. To conjugate the Rhodamine B with the rtPA, the components consisting of Rhodamine B, DCC, and NHS (1:20:20, molar ratio) were first dissolved in a 5 mL of chloroform under vigorous stirring at 1,150 RPM, followed by adding 20 mg of rtPA. The reaction was maintained for 24 hrs. The unreacted rtPA was removed through dialysis. The final products were purified using freeze-drying for 24 hrs and stored at 4 °C until used. **Scale of images: 3 μm.**

|  | Size from TEM Images (nm) |
| --- | --- |
| Superparamagnetic Iron Oxide Nanoparticles (SPIONs) | **10.70 (n=2053)** |

**Supplementary Figure 2.** Synthesized SPIONs through thermal decomposition method. Size analysis using NIH ImageJ software, showing approximately 10.70 nm in TEM images (n=2053).

**Supplementary Figure 3.** Representative XRD pattern of SPIONs nanocrystals, showing the peaks at 2θ at 30.1, 35.4, 42.9, 56.9, and 62.3° in the XRD, assigning to diffraction of the (220), (311), (400), (511), and (440) planes, respectively.

**Supplementary Figure 4.** EDS analysis of rmDPPs. Chemical compositions of rmDPPs within the matrix were carbon (C), iron (Fe), and sulfur (S), indicating that the SPIONs and rtPA were successfully localized in the polymeric matrix.


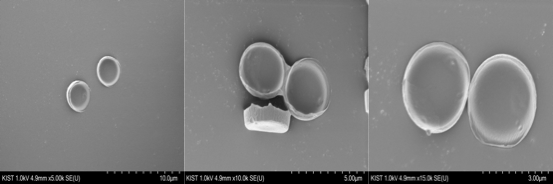


**Supplementary Figure 5.** Additional SEM images of rmDPPs with various resolution (10, 5, and 3 μm, respectively), showing disc shape and uniform size.

**Supplementary Figure 6.** Acoustic system for in vitro and in vivo magneto-sonothrombolysis. **(a)** In vitro acoustic system consisted of unfocused transducer and acrylic water tank (yellow circle: the site that acoustically transparent confocal dish could be mounted). **(b)** In vivo acoustic system contained focused transducer and acoustic housing fabricated using 3D printing techniques (Specification of housing: 10 mm diameter with 13 mm height). **(c-d)** Typical experimental images of in vitro and in vivo magneto-sonothrombolysis.

**Supplementary Figure 7.** Pilot results of enzymatic activity of 0.1, 0.5 and 2 mg of RhoB-rtPA loaded rmDPPs with/without acoustic stimulus. Especially, 2 mg of the rtPA-loaded rmDPPs showed an underestimated enzymatic activity compared to the 1 mg loaded rmDPPs. Threshold line: the enzymatic value of 1 mg loaded rmDPPs with the US.


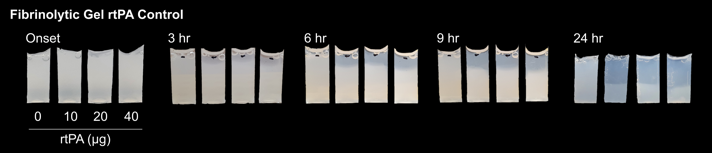


**Supplementary Figure 8.** Determination of rtPA dose in fibrinolytic gel model, indicating that 10 μg of rtPA was sufficient to lyse the gel. Optimized dose of rtPA for fibrinolytic gel: 10 μg.


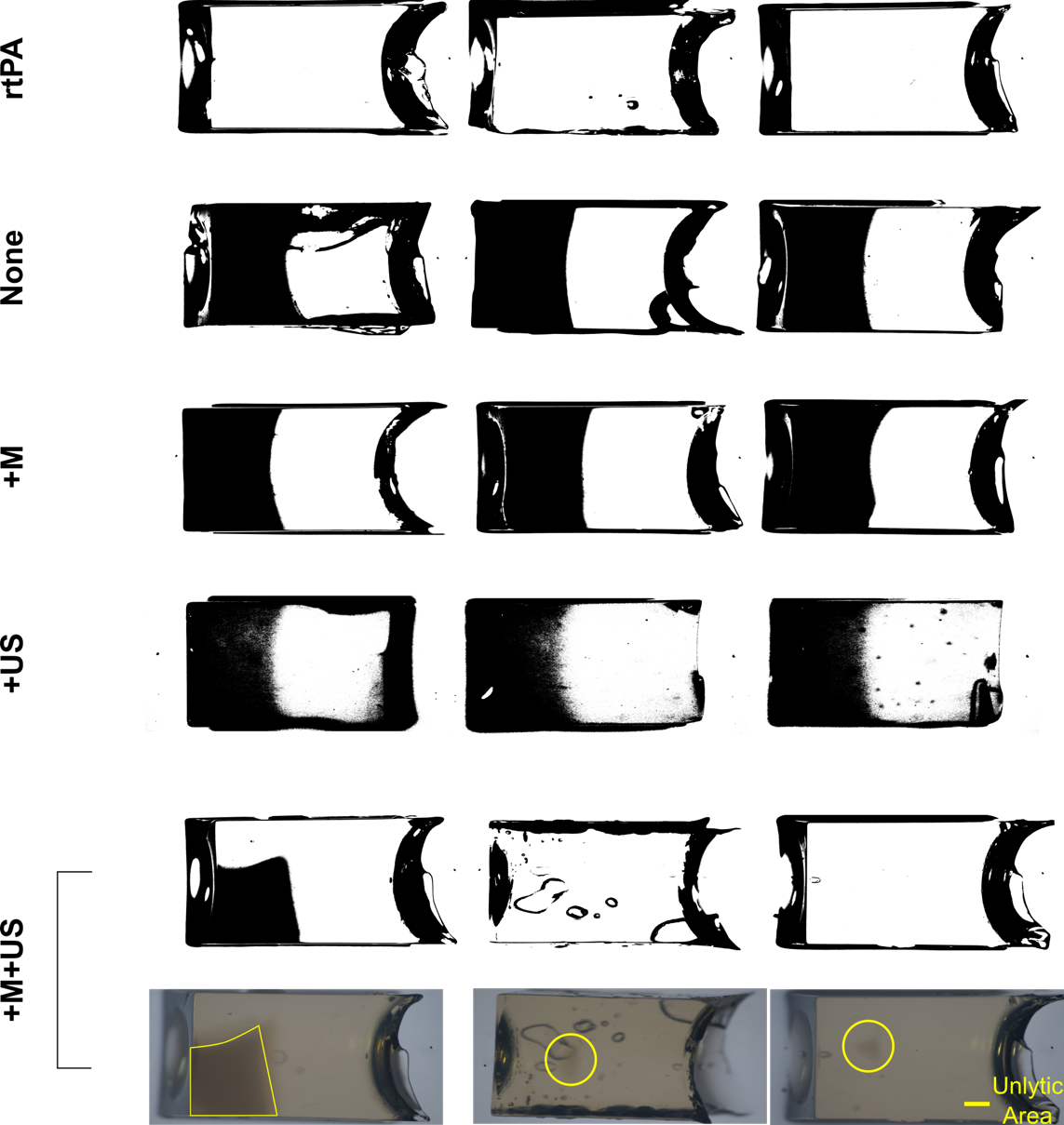


**Supplementary Figure 9.** Analysis of gel fibrinolysis using binary image processing. The fibrinolytic gels in the +M+US group were presented with optical images together since the unlytic areas were not clearly distinguished in the binary images (unlytic area: yellow polygon and circles). Different fibrinolytic patterns were obtained in the +M+US group, showing that unlytic areas were left in the one-side bottom or in the center.

**Supplementary Figure 10.** Determination of the status of in-vitro blood clots. **(a)** Stable blood clots were not reactive to the normal saline, while the saline slightly lysed unstable blood clots. Only stable blood clots were chosen for thrombolysis exper- iments. **(b)** rtPA dose-dependent lysis of blood clots. Overall rtPA-involved groups showed blood clot lysis, and improvement in thrombolysis was observed along with the rtPA dose.

**Supplementary Figure 11.** ***In vivo*** pharmacokinetics of systemically circulating rmDPPs. **(a)** The circulating rmDPPs were originally deposited in the lung without any magnetic field. **(b)** The diminished NIRF signals near to the brain after removal of magnetic field. Precise analysis of ***in vivo*** tracking of the rmDPPs toward the magnetic field was hard due to the relatively high field of view in the IVIS system and high signals expressed in the lung. **(c)** The small bleeding originated from the skin incision did not affect the NIRF signals.

**Supplementary Figure 12.** Investigations of dose-dependent rtPA lytic potential in photothrombotic model at 20 min post- stroke. **(a)** Typical TTC-stained brain slices from the mice that were treated with 300, 150, and 75 μg of rtPA, respectively, showing that the therqpeutic function of rtPA was potentiated along with the dose at 20 min post-stroke. **(b)** Quantification of infarct volume. Administration of 75 μg of rtPA was not effective in alleviating the infarct volume compared to the 0 μg of rtPA, indicating that above 150 μg of rtPA showed potent lytic potential in the photothrombotic model.

**Supplementary Figure 13.** Investigations of dose-dependent rtPA lytic potential in photothrombotic model at 3 hrs post- stroke. **(a)** Typical TTC-stained brain slices from the mice that were treated with 300, 150, and 75 μg of rtPA, respectively, demonstrating that the therpeutic efficacy of rtPA was highly diminished at 3 hrs post-stroke. **(b)** Quantifcation of infarct volume. No statistical differences between 75, 150, 300 μg of rtPA treatment for alleviating infarct, showing that delayed administration of rtPA did not function well.

**Supplementary Figure 14.** Validation of acoustic histological safety using H&E staining, showing no any mechanical and bleeding was observed in overall H&E-stained brain slices.

**Supplementary Figure 15.** Disruption of rmDPPs depending on the sonication duration. Fluorescence images of rmDPPs after **(a)** 0, **(b)** 5, and **(c)** 10 min post-sonication, showing that overall size of particles was reduced along with the duration.

1. ^1^ Biomedical Research Division, Korea Institute of Science and Technology,

   Seoul, Republic of Korea. ^2^ Department of Biomedical Engineering, Yonsei

   University, Wonju, Republic of Korea. ^3^ Divison of Bio-Medical Science &

   Technology, Korea Institute of Science and Technology School, Seoul,

   Republic of Korea. ^4^ KHU-KIST Department of Converging Science and

   Technology, Kyung Hee University, Seoul, Republic of Korea [↑](#footnote-ref-1)
2. * Correspondence: jkey@yonsei.ac.kr; han0318@kist.re.kr [↑](#footnote-ref-2)
